# Boron‐Doped Nano‐Crystalline Coated Carbon Fibers for Phasic Dopamine Sensing

**DOI:** 10.1002/adhm.202503945

**Published:** 2025-12-18

**Authors:** Simon J. Higham, Juan M. Rojas Cabrera, Youngjong Kwak, Lydia Hong, Andre Chambers, Athavan Nadarajah, Sorel E. De Leon, Young Jun Jung, Negin Jalilinejad, Charles Blaha, Dong Pyo Jang, Yoonbae Oh, Kendall Lee, Alastair Stacey, Shaun L. Cloherty, Michael R. Ibbotson, David J. Garrett, Steven Prawer, Hojin Shin, Wei Tong

**Affiliations:** ^1^ School of Engineering RMIT University Melbourne Victoria Australia; ^2^ School of Physics The University of Melbourne Melbourne Victoria Australia; ^3^ Medical Scientist Training Program Mayo Clinic Rochester Minnesota USA; ^4^ Department of Neurologic Surgery Mayo Clinic Rochester Minnesota USA; ^5^ Department of Biomedical Engineering Hanyang University Seoul South Korea; ^6^ School of Biomedical Engineering The University of Melbourne Melbourne Victoria Australia; ^7^ Department of Biomedical Engineering Mayo Clinic Rochester Minnesota USA; ^8^ The Graeme Clark Institute The University of Melbourne Melbourne Victoria Australia

**Keywords:** carbon fiber, dopamine, fast‐scan cyclic voltammetry, nano‐crystalline, neurochemical sensing

## Abstract

Real time, chronic electrochemical detection of neurotransmitters will provide a positive step in the treatment and understanding of neurological disease. However, current electrodes using carbon fibers (CF) fail to perform chronically. While diamond‐based coatings show promise in improving their longevity, achieving a uniform layer of such coatings on CFs is challenging, and the electrodes often lose sensitivity after coating. In this work, a complete and uniform boron‐doped nanocrystalline material grown in a diamond reactor (B‐NCD) was developed to coat CF microelectrodes for neurochemical sensing. The coating was characterized electrically, optically, mechanically, and chemically. The B‐NCD coated CF electrodes were able to detect phasic dopamine at a sensitivity comparable to the most widely used alternatives (uncoated and PEDOT:Nafion coated CFs). During biofouling testing, the B‐NCD coated CF electrodes demonstrated better stability than uncoated CFs and comparable performance to PEDOT:Nafion coated CFs. Moreover, B‐NCD exhibited no signs of degradation during consecutive FSCV applications, while uncoated and PEDOT:Nafion coated CF electrodes degraded significantly over time. Furthermore, the B‐NCD coating supported the survival and development of neurons and astrocytes in vitro, exhibited excellent adhesion and durability during mechanical bending testing, and enabled successful in vivo recording of phasic dopamine release in the rat brain. Overall, B‐NCD coated CFs present as an ideal candidate for chronic, flexible neural implantable electrodes for long‐term neurochemical monitoring.

## Graphical abstract

This uniform coating presents a mix of graphitic and diamond‐like carbon to create a mechanically and electrically robust alternative for the electrochemical detection of dopamine. The electrodes in this work possess the equivalent sensitivity to the state‐of‐the‐art FSCV dopamine detecting electrodes, whilst demonstrating an ability to withstand repeated cycling that would normally result in complete degradation of bare carbon fibers. These electrodes are also effective for use in the brain, with proof‐of‐concept work conducted with electrodes implanted into the brain for biologically relevant dopamine detection. These hybrid electrodes balance the electrochemical robustness of diamond with the mechanical flexibility of ordered graphite structures to provide an exciting step toward the perfect chronic neurochemical sensing electrode.

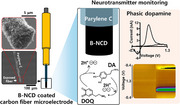

## 1 Introduction

Dopamine is a neurotransmitter involved in various neurological pathways, with functions including learning, memory, reward and pleasure, and motor control [1, 2]. Abnormal dopamine activity in the central nervous system has been found to be associated with many neurological disorders, including neuromotor pathologies like Parkinson's disease and neuropsychiatric pathologies such as schizophrenia, addiction, depression, and anxiety‐related disorders [3, 4, 5]. Therefore, accurate measurement and modulation of this neurotransmitter can provide a unique avenue for deepening our understanding of its pathophysiology and pathogenesis, improving the precision of its diagnosis, and inspiring the development of more effective therapies [6].

Microdialysis is a widely used technique for measuring dopamine levels in the brain [7, 8, 9]. This method involves the insertion of a probe into the brain with a semi‐permeable membrane that allows small molecules, including dopamine, to diffuse into it. Once the dialysate is perfused through the membrane, the solution can then be analysed offline. However, microdialysis probes are quite invasive due to their size, often possessing a diameter >200 µm [10]. Thus, significant tissue damage during probe insertion occurs, limiting spatial resolution and making it unsuitable for long‐term applications. Furthermore, the temporal resolution provided by microdialysis (>1 min) is dependent on the offline analysis process of the analyte [7]. In contrast, electrochemical techniques like voltammetry use smaller electrodes that have been employed for real‐time dopamine sensing [11]. This is possible as dopamine molecules can be electrochemically oxidized and reduced. Such electrode materials include carbon fibers (CF), with a diameter of 7 µm, which can provide higher spatial selectivity with minimal tissue damage, improved safety, and better temporal resolution (milliseconds to seconds) [12].

Fast scan cyclic voltammetry (FSCV) is an electrochemical method commonly employed for measuring stimulation‐evoked (i.e., phasic) levels of electroactive neurotransmitters levels such as dopamine, in the brain [11, 13]. With traditional FSCV for measuring changes in dopamine, a triangle‐shaped waveform (−0.4 to +1.3 V with a sweep‐rate of 400 V/s) is applied to the recording electrode at a repetition rate of 10 Hz. As the potential is swept from the anode and cathode, electroactive molecules adsorbed onto the surface of the electrode, like dopamine, are oxidized and reduced, and the current generated from the redox reaction is measured. Compared to conventional cyclic voltammetry (CV) that typically scans at a rate of 100 mV/s, the faster scan rate used in FSCV allows for both increased temporal resolution and detection sensitivity, both of which are vital for in vivo neurotransmitter detection due to their low concentrations in tissue and the high speed of release [11]. However, due to this rapid scan rate, FSCV also generates a large capacitive background current that must be digitally subtracted, resulting in isolation of the faradic current for the quantification of dopamine [11].

Recording microelectrodes utilizing CF are the most common type of biosensors investigated for FSCV detection of dopamine [12, 14, 15, 16, 17, 18, 19]. CF microelectrodes have surface oxide functional groups that allow for the adsorption of cations, a feature that is vital for electrochemical dopamine detection [12, 20]. However, long‐term, in vivo, FSCV recordings with CF microelectrodes are limited due to two factors. First, biofouling, in which protein and other biomolecules accumulate on the electrode surface after prolonged implantation in the brain, can degrade the performance of the microelectrode by decreasing its sensitivity. Second, CF microelectrodes have limited electrochemical stability and can be gradually etched after repeated FSCV cycling [11, 21, 22]. To overcome these challenges, many groups have explored alterations and enhancements of CFs for dopamine detection [23, 24, 25, 26]. These include surface modifications to increase either sensitivity or the ability of the electrode to perform chronically [23, 24]. For example, conductive polymer coatings such as poly(3,4‐ethylendioxythiophene) (PEDOT) using various dopants have shown a two‐fold increase in sensitivity when compared to uncoated CF [23, 27, 28, 29]. However, these studies have not shown any in vivo improvement in dopamine detection lifetime, and hence further work is required in establishing a long‐term dopamine sensing electrode solution [23, 24, 30].

Other alternatives to CF‐based microelectrodes have been explored, with one of the most promising being utilizing sp^3^‐hybridized carbon material like boron‐doped diamond and nanodiamond [24, 25, 31, 32, 33, 34, 35, 36, 37]. Diamond presents unique properties such as biocompatibility, bio/chemical inertness, and electrochemical stability, making them suitable for many biomedical applications [36, 38, 39, 40, 41, 42, 43]. Diamond‐based coatings also possess a higher resistance to biofouling‐effects, an important characteristic for chronically implanted neural electrodes [44, 45]. Their large electrochemical window makes them more resistant to repeated CV cycling [46]. Diamond films can be deposited on various substrates using chemical vapor deposition (CVD) [47]. When boron or nitrogen is introduced during deposition, the diamond films become conductive [48, 49, 50]. Depending on the crystal size, diamond can be categorized into polycrystalline diamond (crystal size in mm range), nanocrystalline diamond (nm range), and ultra‐nanocrystalline diamond (<5 nm) [51]. Compared to polycrystalline diamond, smaller crystal sizes allow for more conformal coatings on non‐diamond substrates [51]. Boron‐doped polycrystalline diamond has been previously deposited on tungsten electrodes for neurotransmitter sensing [52, 53, 54]. Due to the anti‐biofouling properties and their large electrochemical window, these coated electrodes demonstrated higher stability compared to the conventional CF electrodes for dopamine sensing with FSCV. However, the diamond‐coated electrodes often show a loss of sensitivity and may fail to detect concentrations relevant to the brain [24]. Furthermore, the large diameter of these tungsten electrodes (∼100 µm) restricts their chronic use in vivo due to the adverse tissue response.

Sp^3^‐hybridized carbon materials have previously been grown onto CFs with varying success for different applications [24, 25, 55, 56]. These electrodes aimed to leverage the electrical and chemical benefits of the coating with the small size and flexibility of the underlying fiber. The hardness of nanocrystalline diamond will assist in the implantation of CFs, but can also be a potential failure mechanism of flexible electrodes as a result of the coating cracking [57, 58]. Due to the mechanical difference between the underlying electrode and the coating, delamination can occur as the electrode moves and bends within the tissue. A composite material that presents the electrical, chemical, and biocompatibility of diamond, whilst limiting the mechanical mismatch between the underlying electrodes and the coating would allow for an effective chronically implanted electrode.

**SCHEME 1:**
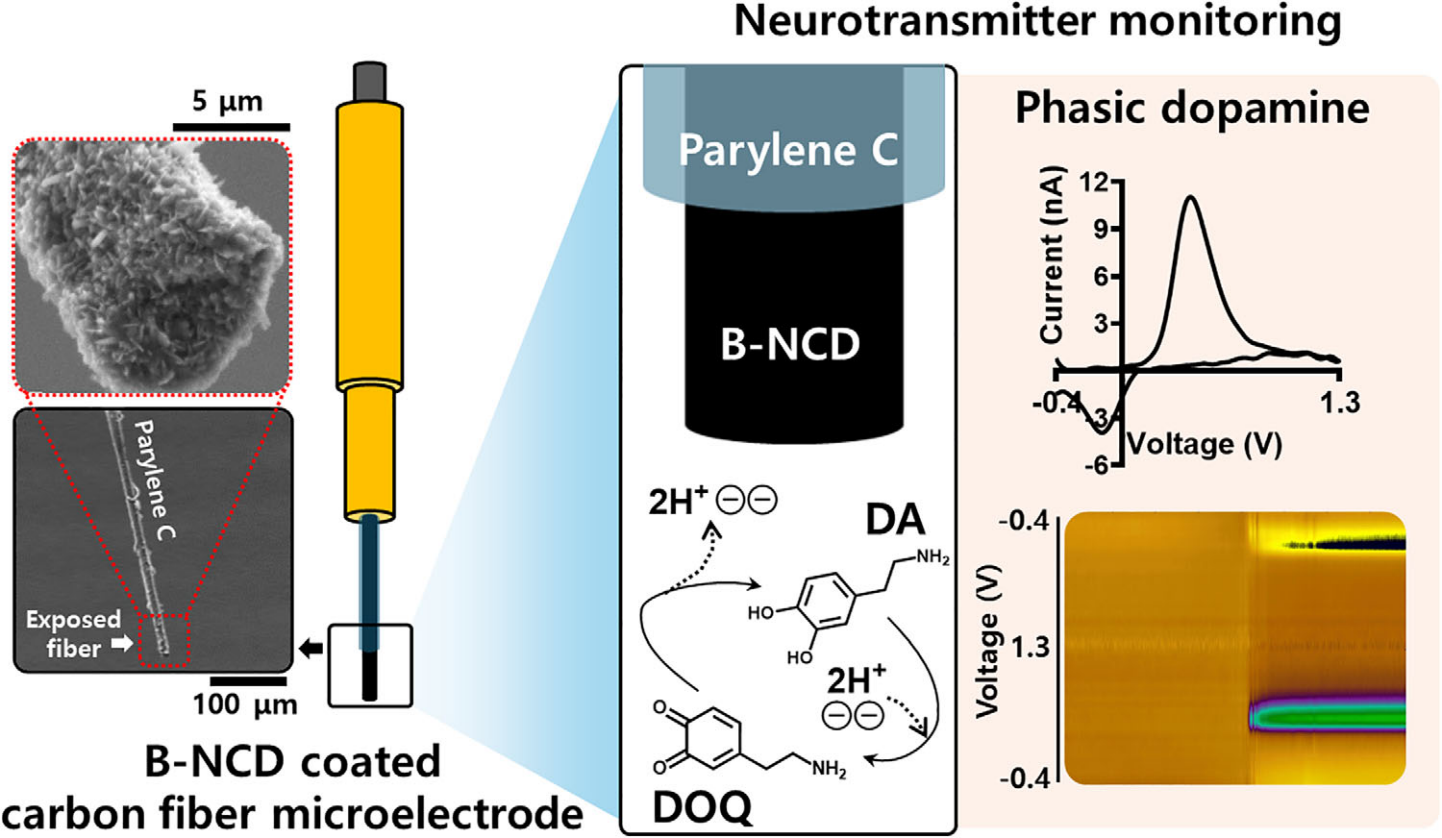
Schematic of B‐NCD coated carbon fiber microelectrode, dopamine oxidation and reduction reaction with the associated voltammogram obtained during dopamine detection measurements.

Here, we report the deposition of a uniform boron‐doped nanocrystalline material in a CVD diamond reactor (B‐NCD) on CFs. The coated fibers have a diameter of <10 µm, retaining the minimal invasiveness of these electrodes. The coating was characterized using a range of techniques including scanning electron microscopy (SEM), Raman spectroscopy, transmission electron microscopy (TEM), and electron energy loss spectroscopy (EELS). Its electrochemical performance was evaluated by measuring phasic dopamine responses with FSCV and comparing stability against uncoated and PEDOT:Nafion coated CFs. Biocompatibility was assessed by culturing primary rat neurons and astrocytes on the B‐NCD surface, while mechanical durability was examined through bending tests to evaluate resistance to delamination. Finally, an in vivo proof‐of‐concept recording was performed to demonstrate the coating's functional applicability for dopamine detection. Collectively, this work underscores the potential of using B‐NCD as a coating on CF for developing a microelectrode for long‐term, chronic neurotransmitter sensing (Scheme 1).

## 2 Materials and Methods

### 2.1 Deposition of B‐NCD on CFs

Achieving optimal deposition of B‐NCD on CF (GoodFellows) surfaces necessitates a preliminary fiber pretreatment. First, amino phenyl functional groups were grafted onto the CF surface using a two‐step electrochemical method and a potentiostat (Gamry Instruments, Warminster, USA), as previously described [59]. An Ag/AgCl and a glassy carbon electrode served as the reference and counter electrodes, respectively. The process involved two distinct solutions. The initial step used an acetonitrile solution containing 0.1 m tetrabutylammonium tetrafluoroborate (Sigma–Aldrich, St. Louis, USA) and 1 mm nitrophenyl diazonium tetrafluoroborate (Sigma–Aldrich, St. Louis, USA). Five CV cycles, between 0.2 and −0.6 V at a scan rate of 200 mV/s, were conducted to deposit a nitrophenyl film on the CF surface [60]. After the deposition, the fibers were rinsed with acetone and deionized (DI) water. For the second step, a solution of 0.1 m H_2_SO_4_ was used. Another set of five cycles was performed, between 0.5 and −1.5 V at the scan rate of 200 mV/s, resulting in the formation of aminophenol groups on the CF surface. A final rinse with acetone and DI water was conducted.

Then, the functionalized CFs were prepared for B‐NCD deposition by seeding with oxygen‐terminated nanodiamonds. The seeding solution was prepared following a protocol previously detailed in the literature [61], containing nanodiamonds at a concentration of 8.47 × 10^7^ particles/ml, with an average particle size of 35–40 nm [62]. The pretreated fibers were immersed in the nanodiamond solution with 0.1 m 1‐Ethyl‐3‐(3‐dimethylaminopropyl)carbodiimide (EDC) and were left to incubate for 24 h. This step was performed within 48 h before the deposition. Post‐incubation, the fibers were rinsed with DI water and subsequently dried using nitrogen gas.

**FIGURE 1:**
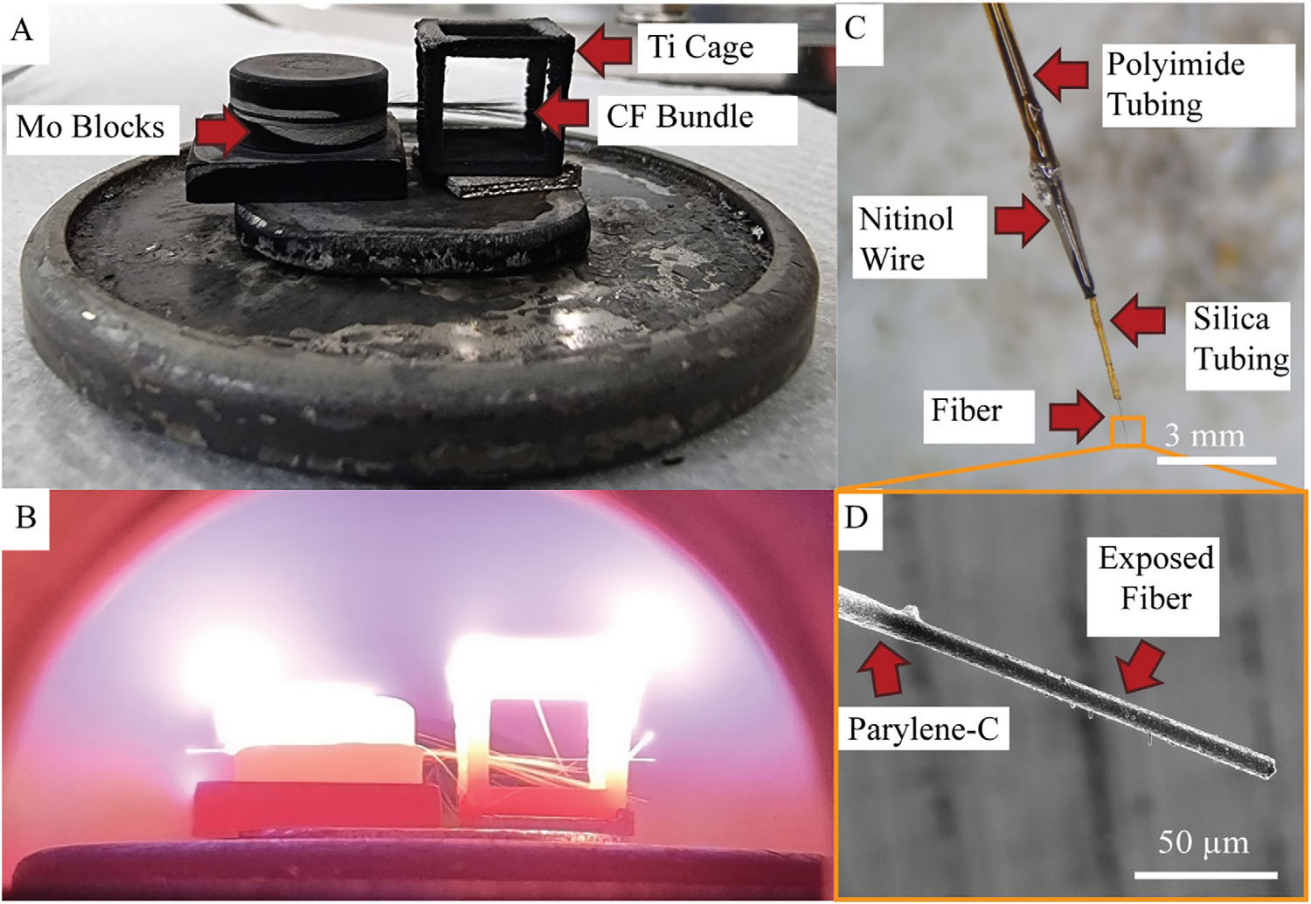
(A, B): The setup used during CVD deposition. CFs were situated within a titanium cage and were held in place with molybdenum blocks post (A) and during growth (B). C. D: CF microelectrode configuration. Individual fibers were connected to nitinol wires and insulated using silica and polyimide tubing (C). D shows the zoom‐in SEM image of a fiber tip. The fiber was coated with Parylene‐C, with only the tip exposed.

The bundle of fibers was then transferred to a microwave plasma‐enhanced CVD diamond reactor, placed on a molybdenum block, and secured between two further blocks (Figure 1A,B). A 3D printed titanium cage was placed over the exposed end of the fibers to allow for more control of the plasma around the fiber bundle. The bundle was spread evenly through the cage to allow for consistent growth on fibers across the bundle as well as to reduce the chance of the growth connecting adjacent fibers. A gas mixture of 94% H_2_, 3% CH_4,_ and 3% Trimethyl boron (TMB 2% boron) was used during the deposition. The deposition time was 60 min using a pressure of 40 torr and a microwave power of 1500 W. Samples were allowed to cool completely under a turbo vacuum, a step crucial for preserving the integrity of the titanium cage.

### 2.2 Electrode Fabrication

Electrodes were fabricated using nitinol wire with polyimide insulation to allow for efficient characterisation. Individual B‐NCD coated CFs were isolated from the bundles, threaded into silica tubing (20 µm inner diameter, 90 µm outer diameter), and secured with an Aryldite Epoxy (Selleys). One end of the fiber was connected to the nitinol wire (Fort Wayne Metals, Fort Wayne, USA) using silver paste (Sigma–Aldrich) (Figure 1C). The paste was baked at 125°C until cured and then covered with a layer of epoxy. About 2 µm of Parylene‐C was deposited using a PDS 2010 LABCOTER 2 Parylene Deposition System to create an insulating layer over the length of the electrodes. To expose the electrode tip, the entire electrode was submerged in DI water with only the required length of the fiber exposed above the surface of the water. A butane torch was applied briefly to remove the Parylene‐C coating and expose the fiber tips (Figure 1D). B‐NCD electrodes used for FSCV measurements were estimated with an exposure length of 200 µm.

Uncoated and PEDOT:Nafion coated electrodes were fabricated by threading individual AS4 CFs (Hexcel, Stamford, USA) into a 5 mm‐length silica tube (20 µm inner diameter, 90 µm outer diameter) and set in place with epoxy. Next, the silica was placed onto a nitinol wire and secured using silver paste. The electrodes were baked at 150°C for 45 min, allowing the silver paste to cure. Polyamide tubing (0.0089″ inner diameter, 0.0134″ outer diameter, 0.00225″ wall thickness; Vention Medical, Salem, USA) was then placed over the electrode and secured with epoxy, allowing 2.5 mm of silica with the CF and 2″ of the nitinol wire (Fort Wayne Metals, Fort Wayne, USA) on the opposite side exposed. A scalpel was used to trim the CF to a length of around 40 µm [63]. For CF electrodes undergoing electrochemical coating, coating was performed with electropolymerization using a potentiostat, (Warminster, USA) in a low‐density PEDOT:Nafion solution, as previously described (−0.8 to 1.5 V, scan rate 100 mV/s for 15 cycles) [23].

Prior to dopamine measurements, all electrodes were placed into Tris solution and stabilized with FSCV using a modified triangle waveform (−0.4 to +1.5 V, scan rate of 400 V/s, and applied at a rate of 20 Hz).

### 2.3 Surface and Chemical Characterization

Physical characterization of the fibers was conducted using scanning electron microscope (SEM), a transmission electron microscope (TEM), EELS, and Raman spectroscopy. SEM images were obtained with a FEI Quanta SEM. TEM images and EELS were produced by a JEOL 2100F Transmission Electron Microscope. TEM images were taken on a cross‐section of a representative B‐NCD coated fiber prepared using focused ion beam (FIB). This sample was welded to a copper TEM sample stage using platinum and then further thinned to 120 nm. A Renshaw InVia Raman with a 532 nm excitation wavelength was also used to understand the chemical properties of the different fibers.

The electrochemical properties of the fabricated electrodes were investigated using a potentiostat (Gamry Instruments, Warminster, USA) with an Ag/AgCl reference electrode and a glassy carbon counter electrode. The capacitance of the electrodes was determined from the electrochemical impedance spectroscopy (EIS) using an R||CPE equivalent circuit model (See Figure S3). This allows the modelling of bioimpedance and electrochemistry by using an equivalent circuit made up of an interpolated capacitor and resistor [64]. This fitting was done via the Levenberg–Marquardt Method to the Nyquists plots using Gamry Framework.

### 2.4 FSCV Measurements

All FSCV measurements described here were performed using the wireless instantaneous neurochemical characterization (WINCS) Harmoni system (Mayo Clinic, Rochester, USA) [65]. Both recording and Ag/AgCl reference electrodes were connected to a remote Harmoni unit that was then paired to a base‐station computer via Bluetooth. Using the accompanying WincsWare software, FSCV waveform parameters were then modified depending on the experimental design.

For dopamine sensitivity testing, the uncoated/ PEDOT:Nafion‐coated/ B‐NCD CF and reference electrodes were placed into a beaker with 30 mL of Tris buffer solution as discussed previously (∼7.4 pH) [66]. A traditional triangle FSCV waveform was applied to the recording electrodes (‐0.4 V ramped up to +1.3 V with a scan rate of 400 V/s applied at a 10 Hz rate). Three boluses of each dopamine concentration tested (1, 1.5, 2, and 2.5 µm) were then injected into the beaker solution. The peak dopamine oxidative current generated for all three boluses was recorded and averaged, resulting in one current value per concentration for each electrode. An identical procedure was performed to produce a calibration curve for the B‐NCD coated CF using dopamine concentrations of 0.1, 0.25, 0.5, 1, 2, and 3 µm.

To test the effects of fouling on the electrodes’ sensitivity, a biofouling environment was simulated using bovine serum albumin (BSA) in TRIS buffer solution at a concentration of 40 g/L [67]. Two separate biofouling experiments were conducted. The first test involved utilizing a modified triangle waveform (−0.4 to +1.0 V, scan rate of 400 V/s) to establish a baseline sensitivity reading for each electrode using a dopamine concentration of 1 µm. Next, the electrode was placed into the BSA solution whilst the modified triangle waveform was continuously applied at a rate of 10 Hz for 4 h in BSA. Electrode sensitivity was subsequently measured at 1, 2, and 4 h in BSA, generating a total of three values per electrode. In the second biofouling test, baseline sensitivity was established for each electrode using a traditional FSCV waveform (−0.4 to +1.3 V, scan rate of 400 V/s, 10 Hz) and 1 µm of dopamine. The electrodes were then submerged in BSA without repeated FSCV applications. After 24 h of incubation in BSA solution, the electrodes’ sensitivity was tested with FSCV using the same waveform parameters (−0.4 to +1.3 V, scan rate of 400 V/s, 10 Hz) and 1 µm of dopamine. This was repeated an additional two times, resulting in the electrodes being submerged into BSA for a sum of 72 h and generating four sensitivity values per electrode.

Longevity testing was also performed by submerging the electrodes in saline (0.15 m NaCl) under repeated FSCV cycling. In this test, repeated FSCV cycling was performed using an altered waveform to more efficiently mimic long‐term chronic neurotransmitter applications (−0.4 to +1.5 V, scan rate of 400 V/s, 60 Hz, up to a simulated period of 108 h). SEM, impedance measurements, and dopamine sensing measurements were obtained to establish the effect of the repeated cycling on the electrodes.

FSCV voltammograms were processed using WINCSWare. Normalized dopamine values in the sensitivity tests were calculated by dividing the background‐subtracted dopamine oxidation peak (post‐dopamine bolus) current value by the maximum pre‐bolus background current measured 10 s before the dopamine challenge. Calibration curve values in Figure S10 were normalized by dividing the background‐subtracted dopamine oxidation peak of each concentration (0.1–2 µm) by the maximum oxidation current generated at the highest concentration tested (3 µm) and expressed as a percentage relative to this concentration.

The limit of detection (LOD) for each CF type (*n* = 5) was calculated using the equation LOD = 3 ^*^ SD/s, where *SD* is the standard deviation of 3 s of background noise measured at the dopamine oxidation potential (+0.6 V) prior to analyte introduction into the buffer solution, and *s* is the slope of the calibration curve generated from dopamine concentrations ranging between 1.0 to 2.5 µm. Figures were created in GraphPad Prism 10 (GraphPad Software, San Diego, CA, USA) and compiled in Microsoft PowerPoint.

### 2.5 Primary Cell Culture

All cell culture procedures adhered to the guidelines outlined by the National Health and Medical Research Council of Australia (NHMRC) and were approved by the University of Melbourne's Animal Ethics and Welfare Committee (Ethics ID: 27269).

Bundles of B‐NCD coated CF were secured onto the surface of coverslips using a biocompatible silicone (WPI KWIK‐CAST(S) Low Toxicity Silicone Adhesive, World Precision Instruments). Sterilization involved a 30‐min incubation in 70% ethanol, followed by three rinses with sterile ultrapure water (MilliQ). Once dried, samples were exposed to UV light for 30 min and subsequently transferred into the wells of 48‐well plates for cell culture. For coating, all samples were incubated with 0.05 mg/mL Poly‐D‐Lysine (PDL; Gibco, Cat#A3890401) for one hour, followed by three washes. PDL‐coated samples were then pre‐incubated in Neurobasal A medium (Gibco, Cat#10888022) for over 24 h before cell seeding.

Primary cortical cells were harvested from postnatal day 0 (P0) rat pups as described in [68]. Following decapitation, heads were transferred into cold Hank's Balanced Salt Solution (HBSS; Sigma–Aldrich, Cat#H9394). Using forceps, skin and skull tissue were carefully removed, and the cerebral cortices from both hemispheres were dissected. Meninges were removed under a dissecting microscope, and cortical tissue was minced using scalpel blades.

Tissue digestion was performed in HBSS containing 250 µg/mL trypsin and 10 µg/mL DNase I (Roche, Cat#11284932001) for 20 min at 37°C. To terminate the enzymatic digestion, a defined trypsin inhibitor in HBSS, also containing 10 µg/mL DNase I, was applied. The resulting cell suspension was obtained through trituration using a P1000 pipette, followed by centrifugation, removal of the supernatant, and resuspension in the cell culture medium. The culture medium consisted of Neurobasal A supplemented with 2% B‐27 (Gibco, Cat#17504001), 2 mm GlutaMAX (Gibco, Cat#35050061), and 100 mg/mL penicillin‐streptomycin (Gibco, Cat#15140122). On day 0, cells were seeded at a density of 100,000 cells per well on both the sample and the coverslip controls using medium supplemented with 5% fetal bovine serum (FBS; Gibco, Cat#A3161001). Cultures were maintained at 37°C in a humidified 5% CO_2_ incubator. Medium changes were performed by replacing half the volume with fresh medium (without FBS) on day 1 and subsequently twice weekly.

Cellular morphology was assessed after fixation and immunostaining. On day 7, cells were fixed with 4% paraformaldehyde (PFA) for 10 min, followed by a 10‐min incubation in cold methanol (−20°C). Samples were then rinsed three times with phosphate‐buffered saline (PBS). Neurons and astrocytes were identified via immunostaining using cell‐type‐specific markers. Fixed cells were incubated with a primary antibody solution containing mouse monoclonal anti‐βIII tubulin (1:1000, Abcam, Cat#ab78078) and chicken polyclonal anti‐GFAP (1:1000, Abcam, Cat#ab4674), diluted in a blocking buffer composed of 2% goat serum and 2% fetal bovine serum (FBS) to reduce nonspecific binding. After 30 min of incubation at room temperature, the primary antibody solution was removed, and the cells were washed three times for 10 min each with PBS. Secondary antibodies, Alexa Fluor 488 goat anti‐mouse (1:1000, Invitrogen, Cat#A11001) and Alexa Fluor 594 goat anti‐chicken (1:500, Abcam, Cat#ab150176), were diluted in the blocking buffer and applied for 30 min at room temperature, followed by three additional PBS washes.

Immunostained cells were imaged at randomly selected regions on the fibers using a confocal microscope (Olympus FV1200) equipped with 473 and 543 nm excitation lasers and a Nikon Plan Apo 20× objective lens (0.75 numerical aperture). Image processing and analysis were performed using Fiji (ImageJ).

### 2.6 Mechanical Testing

Mechanical testing was performed to evaluate the effects of the B‐NCD coating on the mechanical properties and durability of CF electrodes. A Mark‐10 Series 7 Force Gauge was used to measure the forces exerted on the electrodes. The fibers were attached to home‐made test equipment in two configurations, using epoxy. The first setup involved a fiber being attached to the holder at one end, with the free end being pressed against a solid surface to determine damage when the fiber buckled to mimic neural insertion failure. The second involved a three‐point bend test, where the fiber was fixed on both ends and the force was applied to the middle of the fiber until mechanical failure. A linear manipulator was used to control the movement during testing to bend the fibers against the test gauge at a speed of 50 µm s^−1^. A microscope camera was used to visualize the degree of bending of the fiber before failure. Fibers were imaged after testing using SEM to evaluate any delamination or damage to the coating after bending.

### 2.7 In Vivo FSCV Validation

For in vivo feasibility testing, a B‐NCD CF was implanted into the brain of a male Sprague‐Dawley rat (300 g; Envigo, Indianapolis, USA) to record stimulation‐evoked dopamine release. The animal was housed in an AALAC‐accredited vivarium in a plastic cage under 12‐h light/dark cycles, 21°C ambient temperature, 45% humidity, and ad libitum access to water and food. On the day of surgery, the animal was anesthetized with urethane (1.5 g/kg, i.p.) and placed into a stereotaxic frame (Kopf Instruments, Tujunga, USA). Two trephine holes were drilled into the skull: one for the placement of the BNCD CF into the nucleus accumbens (AP: +1.2 mm, ML: ±2 mm, DV: 6–7 mm) and a second for the placement of a concentric stimulating electrode into the ventral tegmental area (AP: −5.1 mm, ML: ±0.9 mm, DV: 8–9 mm). A third burr hole was made to insert an Ag/AgCl reference electrode into the contralateral cortex (DV: 1–2 mm). Target coordinates are all relative to bregma and were derived from the Paxinos and Watson Rat Brain Atlas [69].

Stimulation‐evoked dopamine was recorded with FSCV (−0.4 to +1.3 V, scan rate of 400 V/s, 10 Hz) after applying short, charge‐balanced bursts (60 Hz, 2 ms pulse width, 0.3 mA amplitude; 3 s duration) of electrical stimulation to the ventral tegmental area. Changes in phasic dopamine levels were recorded at baseline, with 0.9% normal saline (1 mL/kg, i.p.), and after the injection of the selective dopamine reuptake inhibitor GBR 12909 (20 mg/kg, i.p.). All aspects of animal care and handling are adhered to the NIH Guide for the Care and Use of Laboratory Animals (Department of Health and Human Services, NIH Publication No. 86‐3, revised 2011), and the experimental procedures detailed in this study were approved by the Mayo Clinic Institutional Animal Care and Use Committee.

### 2.8 Statistical Analysis

Data are presented as mean ± standard error of the mean (SEM), with statistical significance defined as *ns* = non‐significant, *p* <0.05 (^*^), *p* <0.01 (^**^), and *p* <0.001 (^***^) unless otherwise noted. All statistical analyses were performed in Prism 10. Two‐way Anova, paired and non‐paired *t*‐tests were performed to compare the significance of the difference between samples. Sample numbers as well as the test performed are stated with each result.

## 3 Results and Discussion

### 3.1 Surface Characterization

**FIGURE 2:**
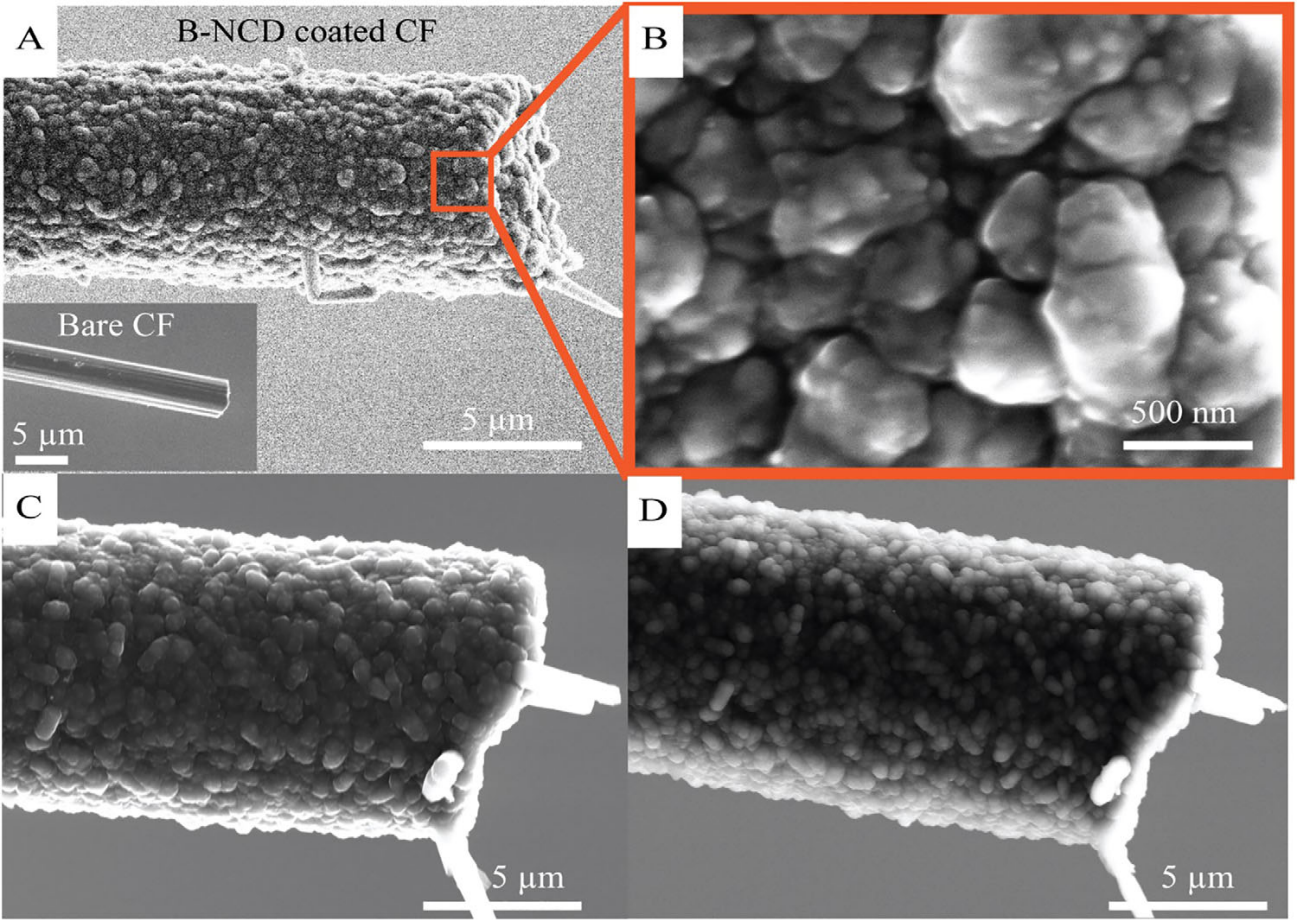
(A,B): SEM images of a CF coated with B‐NCD. The SEM image in (A) shows the uniform coating of B‐NCD, with grain sizes of deposited materials as small as 50 nm (B). (C,D): SEM images of B‐NCD coated fiber before Parylene‐C coating and after Parylene‐C deinsulation using a butane torch. The SEM images show that the coating was intact before (C) and after (D) Parylene‐C coating and deinsulation.

Prior to surface characterization, all fibers were fabricated into electrodes, as outlined in the Section 2.2. The B‐NCD coated fibers were attached onto nitinol wires, coated with Parylene‐C, and subsequently had their tip insulation removed using a fire de‐insulation technique, with an example shown in Figure 1C,D. SEM imaging (Figure 2A) reveals the uniform deposition of B‐NCD on the CFs. The thickness of these fibers increased from 7.0 to 9.0 µm, indicating a B‐NCD coating thickness of 1 µm. Complete coverage was observed on the fiber, with consistent coatings extending onto its tip. As shown in Figure 2B, the deposited coating had grain sizes as small as 50 nm. There was also evidence of carbon nanowall growth within the bundle of CFs (Figure S1). These structures were often seen on fibers that were not uniformly coated with the nanocrystals, indicating they formed during the early stages of B‐NCD growth. This is consistent with previous work on the deposition of boron‐doped diamond on CFs [56]. This work focused on the fibers with nanocrystalline structured coatings, and the carbon nanowall coatings were not studied here. Only electrodes with complete, uniform, and nanocrystalline structured coatings were selected for use in the rest of this study.

The fabrication protocol employed here aligns with established methods for fabricating uncoated CF electrodes [70, 71]. A critical consideration was whether the de‐insulation process could potentially damage the integrity of the B‐NCD coating. To address this, SEM imaging was conducted on the same fibers, both before Parylene‐C coating and after subsequent Parylene‐C deinsulation. As shown in Figure 2C,D, the coating remained intact post‐deinsulation with only minimal surface alteration detected, suggesting the process preserved the B‐NCD coating's structural integrity.

### 3.2 Chemical Characterization

**FIGURE 3:**
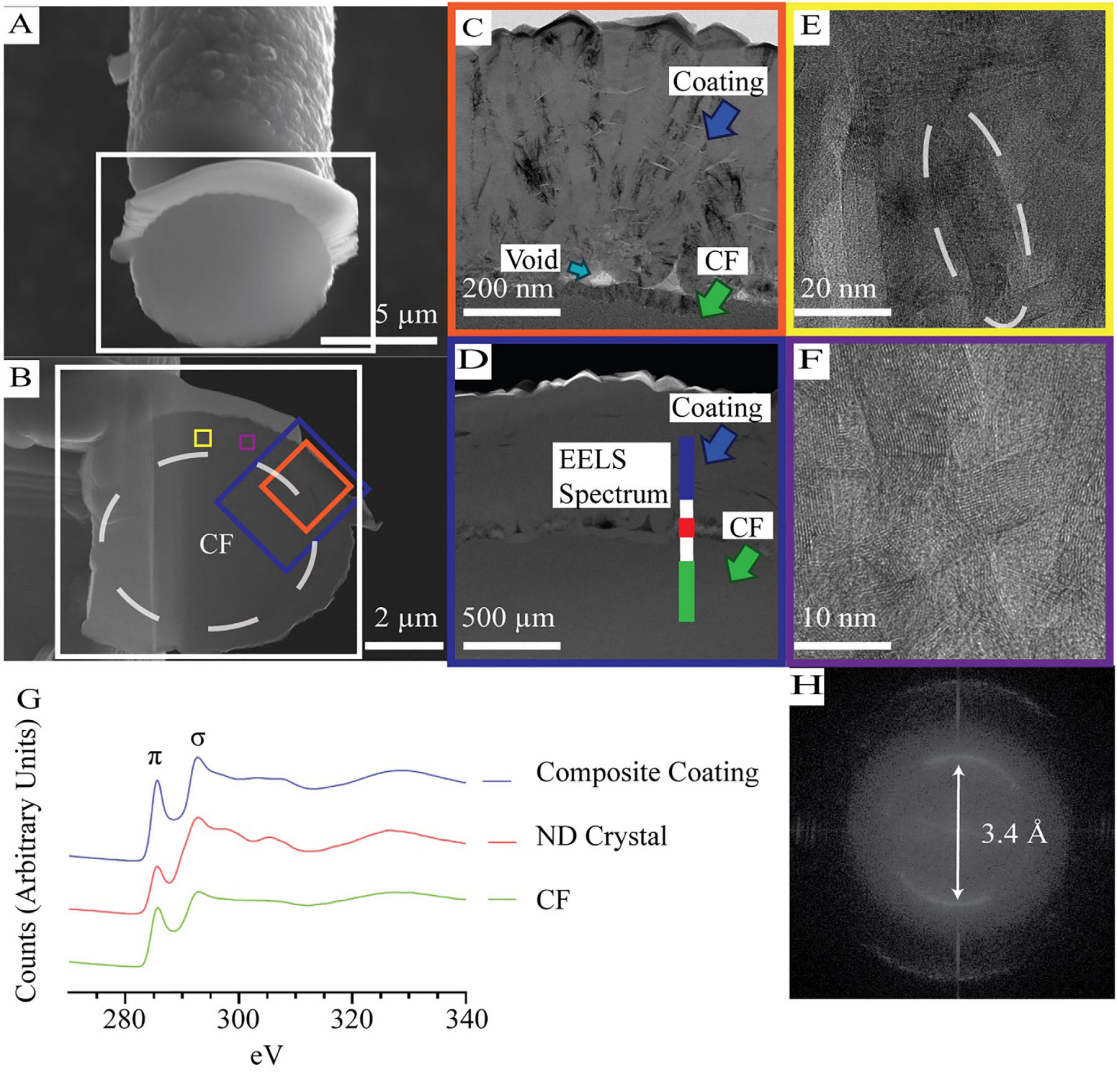
SEM images of the cross‐section of a B‐NCD coated CF sample (A,B). This 120 nm thick cross‐section was prepared using FIB for TEM imaging. TEM images of B‐NCD coated CF (C–F). Boxes of colors indicate the location of different images taken in B. In C and D, green, dark blue and light blue arrows indicate the CF core, the coating, and the void, respectively. E shows the presence of nano‐wall like structure (dashed white) graphite in the coating. F shows nano sized crystal structures of graphite as small as 10 nm. G shows the EELS spectrum obtained from D, with colors indicating the position on the sample where the spectra were taken. These include the bulk composite coating (Blue), nano‐diamond (ND) seed layer (Red) and CF (Green). The diffraction pattern H, indicating the d‐spacing of TEM crystal structure of F.

TEM characterisation was performed to study the crystal structure of the B‐NCD coating [72]. A coated CF sample was mounted, and a cross‐section near the end of the fiber was prepared with a thickness of 120 nm using FIB (Figure 3A,B). We zoomed in on the interface between the CF core and the coating. Consistent with the SEM image (Figure 2B), crystals with sizes as small as 10 nm are visible in the coating layer, as shown in Figure 3F, with small voids observed at the junction between the CF and coating (Figure 3C). This is likely due to the larger crystals growing outward from the CF surface. The size and presence of these voids were low, possibly attributing to the nanodiamond seeding technique employed in this work. Higher magnification TEM images (Figure 3E,F) reveal a mixture of nanometer‐sized graphitic crystals and graphitic nano‐wall structures within the coating, consistent with previous publications [55, 73, 74, 75]. The diffraction pattern produced from the fast Fourier transform (FFT) of these TEM images shows the d‐spacing of the crystal structure to be 3.4 Å. This is consistent with previous work on highly ordered graphitic material [76]. The presence of the carbon‐nanowall within the coating aligns with the growth mechanics reported in earlier work on boron‐doped diamond grown on CF [56]. This work proposed that the initial growth stages of boron‐doped diamond on carbon fiber begin with a carbon nanowall layer growing from the seeding layer. This is followed by nm and later µm scale diamond crystal formation. Our TEM images confirm the formation of a composite material within the bulk of the coating, leading to potentially interesting and tunable mechanical properties.

The EELS spectra collected from different regions, as shown in Figure 3D, showed distinct features (Figure 3G). The CF section (green) showed mostly amorphous carbon as indicated by the smooth curve past 300 eV with an isolated large *π* peak at 285 eV [77]. At the intersection between the CF and the coatings, the EELS spectra (red) showed distinctive sp^3^ carbon features, with a proportionally lower peak at 285 eV (sp^2^ peak) compared to the 302 eV σ (sp^3^) peak. Additionally, the presence of the σ peak at 310 eV is an indication of sp^3^ bonded carbon [78]. This provides further evidence of the successful nanodiamond seeding of the CF. In comparison, the bulk coating measurement (blue) shows evidence of the presence of highly ordered graphite within a sp^2^/sp^3^ hybridized structure with more prominent sp^2^ features.

**FIGURE 4:**
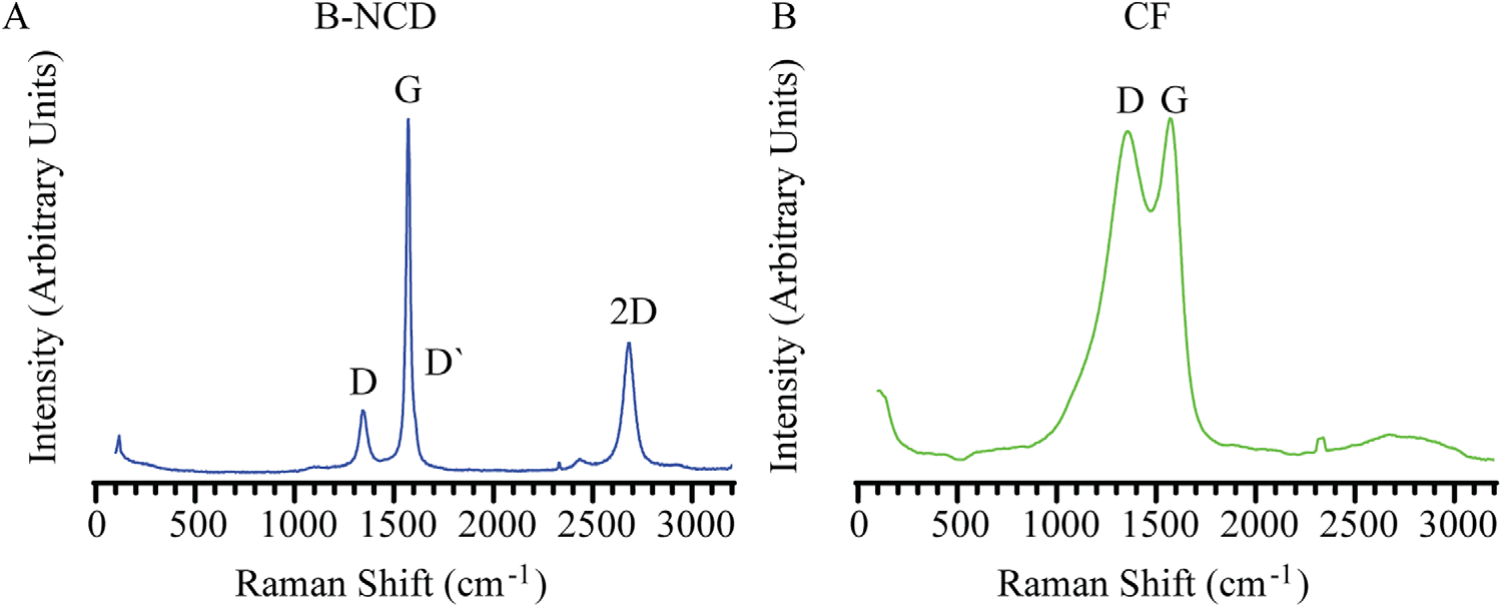
Raman spectra of a B‐CND coated fiber after Parylene‐C deinsulation (A) and an uncoated CF (B) The D and G peaks are representations of sp^2^ bonded carbon. The D` and 2D peaks are evidence of highly ordered graphitic bonding.

To further understand the chemical composition of the coating, we performed Raman spectroscopy on both B‐NCD coated and uncoated electrodes (Figure 4), which is a powerful technique for characterizing the structure of carbon materials. The Raman spectra remained consistent across the coated samples tested. Both B‐NCD coated and uncoated samples exhibit distinct sp^2^‐bonded carbon peaks, including D (at 1350 cm^−1^) and G‐peaks (at 1580 cm^−1^) [79]. The B‐NCD coated samples also contained a D` peak (at 1615 cm^−1^), known to represent ordered graphite, which may indicate the presence of carbon nanowalls within the growth [77]. Quantitative assessment was performed by calculating the normalized intensity ratio of the G‐ to D‐peaks for each sample. After the coating, there was a notable increase in this ratio, from 1 to 2.92 ± 0.64, suggesting an augmented presence of ordered sp^2^‐bonded carbon within the coating matrix, as evidenced by the enhanced G‐peak. We did not observe the presence of the typical diamond peak (at 1332 cm^−1^) from any of the samples. Raman at the illumination used is more sensitive to sp^2^ than sp^3^ carbon, showing a layer with highly ordered graphite formed after the growth [80]. Note that Raman spectroscopy was performed before Parylene‐C coating and after fire deinsulation on the same samples; however, no difference was observed.

### 3.3 Electrical Characterization

**FIGURE 5:**
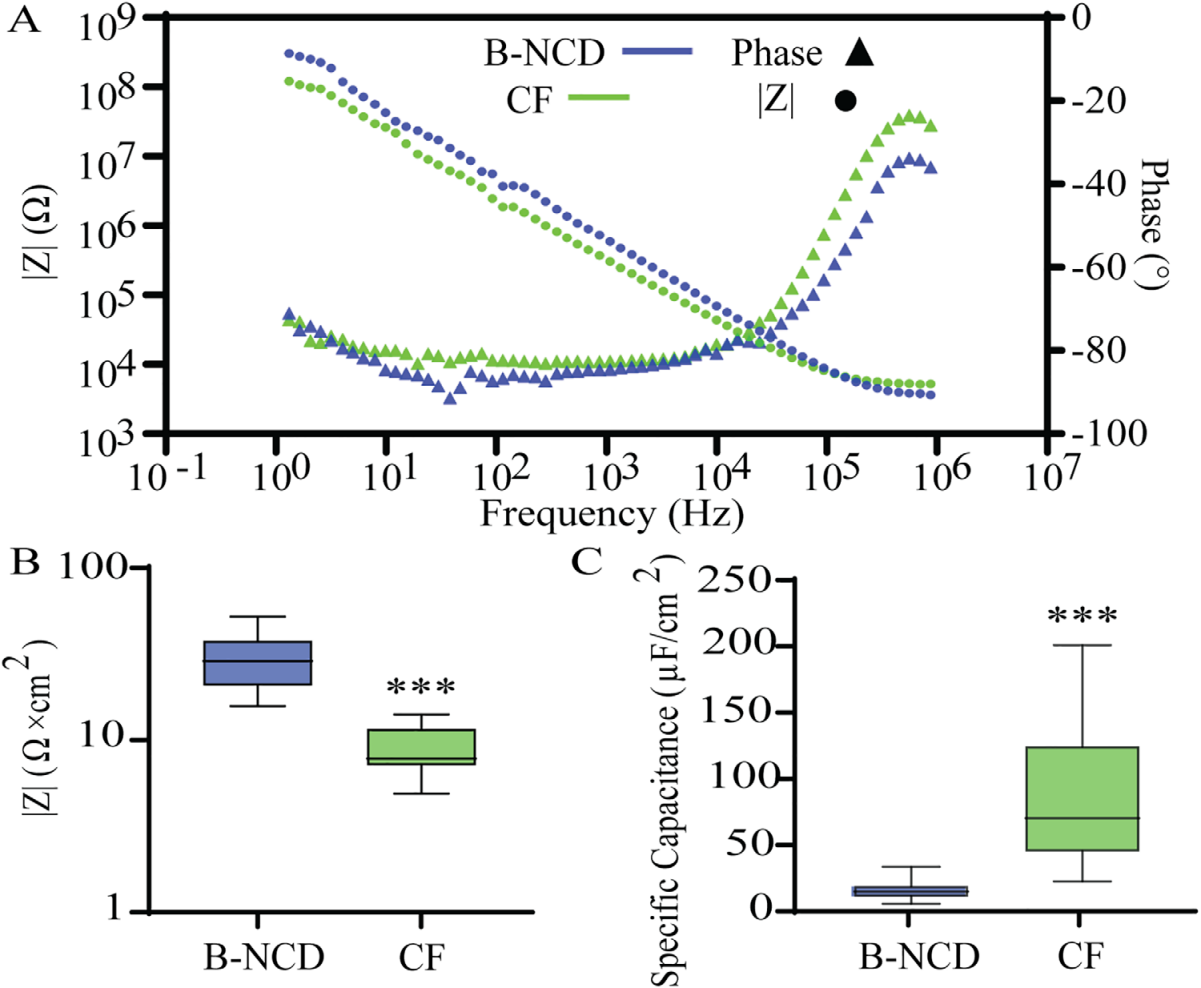
Representative Bode Plot of B‐NCD and CF electrodes, showing the impedance magnitude |Z| and phase angle (°) (A). Impedance magnitude at 1 kHz (B) and Specific Capacitance (C) of electrodes with or without B‐NCD coating (*n* = 12, ^****^ *p*<0.001) extracted from fitting using a Randles circuit model (Figure S3).

Prior to testing the electrodes for dopamine sensing, we also performed electrochemical characterisation of the electrodes. Figure 5A shows the representative EIS spectrum of B‐NCD coated and uncoated CF electrodes. Figure 5B shows the average impedances at 1 kHz of B‐NCD coated and uncoated CF electrodes. The normalized 1 kHz impedance for the B‐NCD and CF electrodes was 32.09 ± 11.09 and 8.95 ± 2.91 Ω × cm^2^ respectively (*p*<0.0001, *n* = 12). These results further indicate the potential presence of sp^3^ carbon within the surface of the B‐NCD coating, as previous work on graphite‐coated CFs produced lower impedance values [62]. This was also consistent with the difference seen on electrodes fabricated from fibers mostly coated in carbon nanowalls from within the growth bundle, as carbon nanowall coating resulted in lower impedance than B‐NCD coatings (Figure S2). Using an equivalent circuit fitting from the EIS plots, we extracted the double‐layer capacitance of the B‐NCD and CF electrodes (Figure S3). The specific capacitance of the B‐NCD and uncoated fibers was 16.1 ± 6.8 and 93.9 ± 39.0 µF/cm^2^, respectively (*p*<0.0001, *n* = 12) (Figure 5C). This difference in conductivity and capacitance between B‐NCD and uncoated CF necessitated longer exposed lengths for the B‐NCD coated electrodes to achieve comparable background currents in the FSCV measurements described below.

### 3.4 Dopamine Sensing Using FSCV Measurements

**FIGURE 6:**
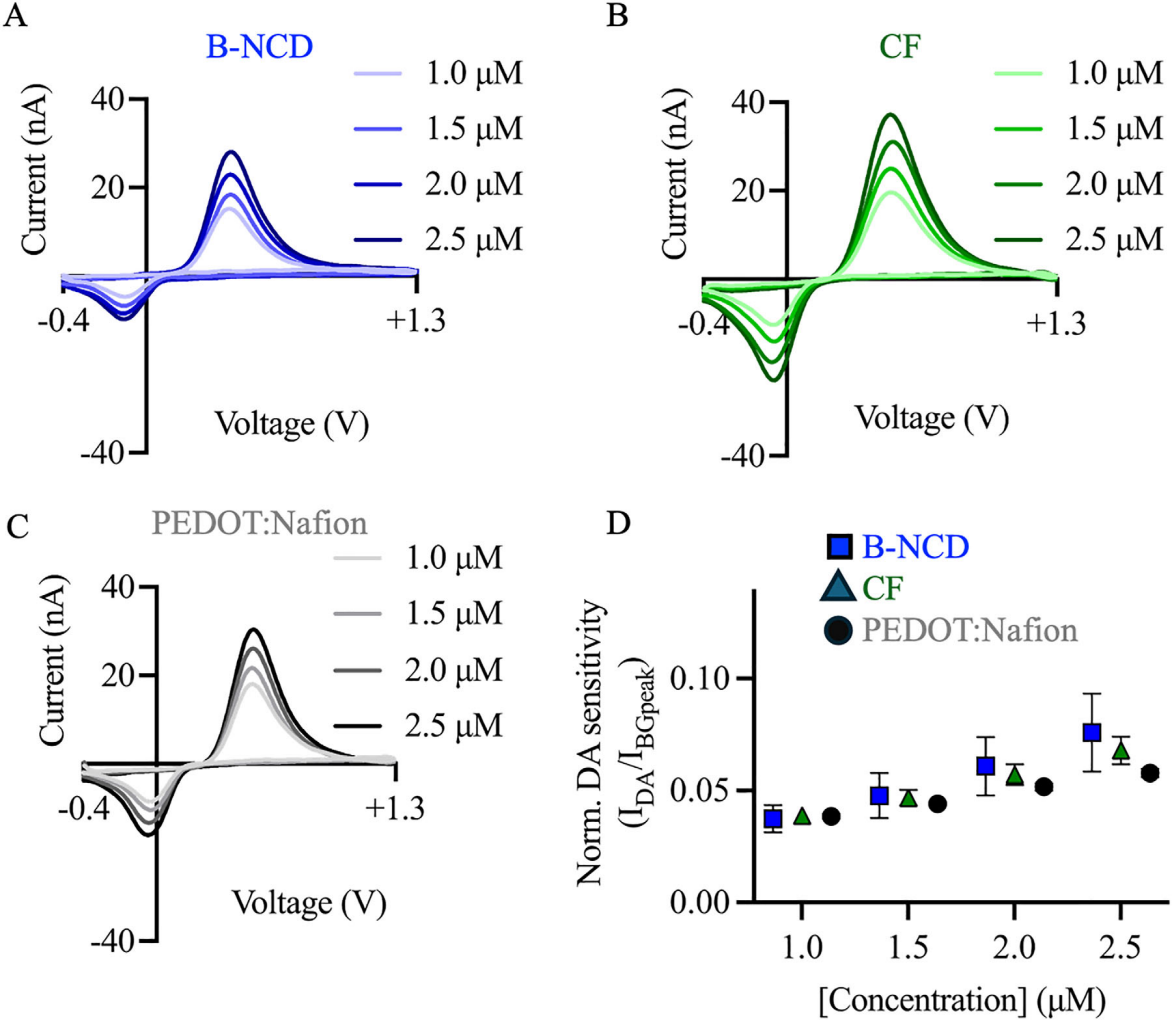
Dopamine sensitivity measurement with B‐NCD, uncoated, and PEDOT:Nafion coated CF using FSCV. Representative voltammograms depicting non‐normalized current response for dopamine concentrations of 1.0–2.5 µm (A, B, C). Dopamine oxidation peak current for each dopamine concentration tested for each of the three electrode groups (blue square = B‐NCD coated CF, green triangle = uncoated CF, black circle = PEDOT:Nafion coated CF). Values were normalized by dividing the peak oxidation value of the voltammogram by the peak value of the background current (*n* = 6/electrode type) (D).

To evaluate the feasibility of B‐NCD coated CF for measuring neurotransmitters using electrochemical techniques, triangular waveform FSCV (−0.4 to +1.3 V, scan rate of 400 V/s, 10 Hz) was employed to obtain background currents. Background subtracted voltammograms for dopamine at concentrations of 1.0–2.5 µm were recorded (Figure 6A–C). This concentration range was chosen to confirm the linear relationship between concentration and current response consistent with previous studies [11, 81, 82]. Dopamine signals for uncoated CF and PEDOT:Nafion coated CF were also obtained to compare the normalized dopamine sensitivity (Figure 6D). The B‐NCD coated CF electrode showed dopamine oxidation within the potential range of +0.4 to +0.5 V (Figure 6A). To ensure similar background currents across all electrodes, we used a 200 µm exposure length for B‐NCD coated electrodes, but 40 µm for uncoated and PEDOT:Nafion coated CF electrodes. The longer exposure length for B‐NCD electrodes helps compensate for the higher impedance and lower electrical conductivity of B‐NCD relative to uncoated CFs. To further account for differences in electrode surface area resulting from manual fabrication variability as well as the corresponding background current magnitudes, the dopamine oxidation peak currents were normalized by the background peak currents (Figure 6D). The normalized dopamine sensitivities at concentrations of 1/1.5/2/2.5 µm for B‐NCD coated CF, uncoated CF, and PEDOT:Nafion coated CF showed no significant difference (Two‐way ANOVA; DA 1 µm, *p* = 0.1111; 1.5 µm, p = 0.3000; 2 µm, *p* = 0.1669; 2.5 µm, *p* = 0.3453). The limit of detection (LOD) for the B‐NCD, uncoated, and PEDOT:Nafion coated CF were calculated to be 0.039, 0.046, and 0.042 µm, respectively. This result indicates that the B‐NCD coated CF electrode exhibits similar performance to both uncoated CF and PEDOT coated CF in the electrochemical detection of dopamine using FSCV. We do not expect that the changes in electrode length required for the ideal background current would impact the performance of the B‐NCD electrodes for in vivo measurements. A length of 200 µm would still allow for the electrodes to sit entirely within dopamine‐rich brain areas, such as the ventral tegmental area and nucleus accumbens, which span depth ranges of up to 500 µm [83].

### 3.5 Stability Assessment

**FIGURE 7:**
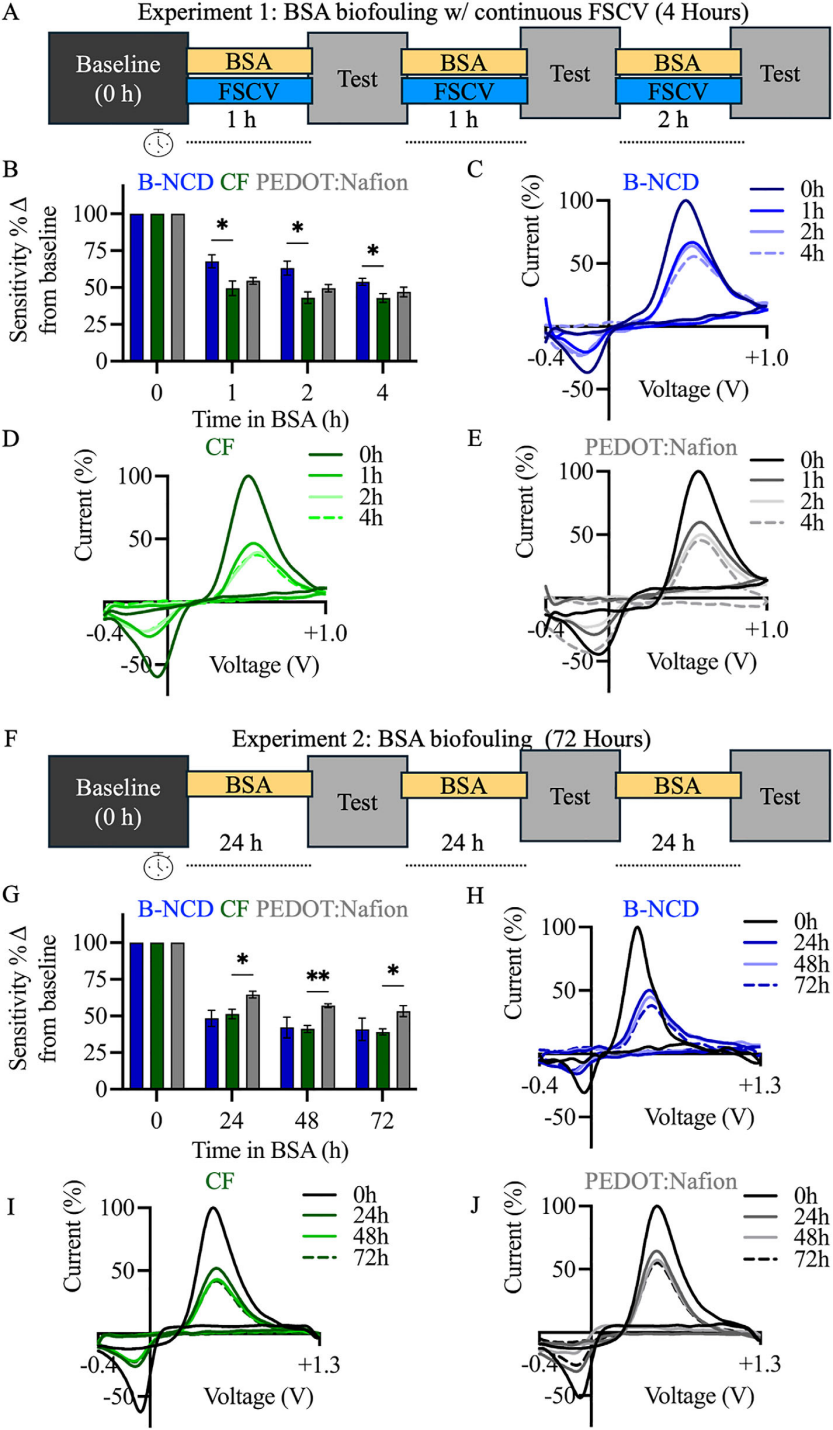
Evaluation of biofouling properties for B‐NCD, uncoated, and PEDOT:Nafion coated CFs. (A, F) Schematic showing experimental design of the two distinct biofouling tests. (B) Normalized changes in dopamine sensitivity at baseline and following exposure to BSA with continuous FSCV waveform application across each electrode type (blue bar = B‐NCD CF, green bar = uncoated CF, grey bar = PEDOT:Nafion coated CF) (*n* = 6/electrode type). Representative background‐subtracted voltammograms for 1 µm of dopamine in Tris buffer obtained at 0, 1, 2, and 4 h of BSA fouling for (C) B‐NCD coated CF, (D) uncoated CF, and (E) PEDOT:Nafion coated CF. (G) Normalized changes in dopamine sensitivity at baseline and after BSA exposure without continuous FSCV waveform application across each electrode type (n = 6/electrode type). Representative voltammograms for 1 µm of dopamine in Tris buffer obtained at 0, 24, 48, and 72 h of BSA fouling for (H) B‐NCD coated, (I) uncoated, and (J) PEDOT:Nafion coated CF.

To investigate the non‐specific protein adsorption characteristics of the B‐NCD coated CF electrode, two separate biofouling experiments were conducted. In the first test, FSCV (−0.4 to +1.0 V, scan rate of 400 V/s, 10 Hz) was continuously applied to the electrodes whilst they were submerged in a high‐concentration BSA solution for 0, 1, 2, and 4 h (Figure 7A). A lower potential range (+1.0 V instead of +1.3 V) was selected to focus on biofouling effects, as higher potentials are known to activate and renew the electrode surface, potentially masking fouling‐related changes. To quantify the degree of fouling, the dopamine oxidation peak current for 1 µm of dopamine before the BSA fouling procedure was set as 100% for normalization (Figure 7B). The BSA fouling experiments were also conducted with uncoated CF (Figure 7D) and PEDOT:Nafion coated CF (Figure 7E) under the same conditions. After the 1‐h fouling session, the B‐NCD coated CF had a 32.2% reduction in dopamine sensitivity. In contrast, the uncoated CF's sensitivity fell by 50.5% while the PEDOT:Nafion coated CF exhibited a 45.5% decrease from baseline. After 2 h in BSA, the B‐NCD coated CF demonstrated a 36.8% reduction in dopamine sensitivity from baseline. Uncoated CF and PEDOT:Nafion coated CF sensitivity fell by 56.9% and 50.4%, respectively. Finally, after 4 h in BSA, the B‐NCD coated CF's sensitivity decreased by 46.1%, while the uncoated CF and PEDOT:Nafion coated CF saw their sensitivity reduced by 57.1% and 52.9%, respectively.

A two‐way ANOVA was performed and revealed that there was a statistically significant interaction between the effects of CF type and time in BSA on dopamine sensitivity (F(6, 45) = 4.8, *p* = 0.0006). Post‐hoc Tukey's comparisons further revealed that the B‐NCD CF had a significantly lower reduction in sensitivity compared to uncoated CF electrodes at 1 h (*p* = 0.0477), 2 h (*p* = 0.0217), and 4 h (*p* = 0.0420) but no significant difference compared to the PEDOT:Nafion coated electrodes. These results suggest that with the application of continuous FSCV to the CF, the B‐NCD electrode has superior anti‐biofouling properties compared to uncoated CF electrodes, and a similar level of resistance to fouling as the PEDOT:Nafion coated CF.

In the second biofouling test, B‐NCD‐coated electrodes were immersed in BSA for a total of 72 h without repeated FSCV applications. Similarly, redox currents were normalized as previously described to quantify sensitivity changes tested (Figure 7E). However, unlike the previous experimental design, FSCV with +1.3 V was used only at baseline and at 24‐h intervals to assess changes in sensitivity with 1 µm of dopamine (Figure 7H). The same protocol was applied to uncoated CFs (Figure 7I) and PEDOT:Nafion coated CFs (Figure 7J). After 24 h in BSA, the B‐NCD coated CFs exhibited a 51.6% reduction in dopamine sensitivity from baseline. In comparison, the uncoated and PEDOT:Nafion coated CFs showed reductions of 48.6% and 35.4%, respectively. At 48 h, the B‐NCD electrodes demonstrated a 57.8% decrease in sensitivity and ultimately reached a total loss of 59.1% at 72 h. Uncoated CFs showed similar declines in performance, with sensitivity reductions of 58.8% at the 48‐h time point and 60.8% after 72 h. The PEDOT:Nafion coated CFs maintained the highest sensitivity across all time points, with losses of 35.4% at 48 h and 46.7% after 72 h.

Similarly to the previous biofouling test, a two‐way ANOVA revealed a statistically significant interaction between the effects of CF type and time in BSA on dopamine sensitivity (F (6, 36) = 2.8, *p* = 0.0219). Post‐hoc analysis with Tukey's multiple comparisons revealed no significant differences in sensitivity between the B‐NCD coated CF and uncoated or PEDOT:Nafion CF during any time point. However, there were significant differences between the uncoated and PEDOT:Nafion coated CF after 24 (*p* = 0.0315), 48 (0.0029), and 72‐h (p = 0.0364) in BSA. The differing outcomes between the two biofouling tests provide useful insight into the performance of B‐NCD coated CF electrodes. The results indicate that these electrodes perform well under continuous FSCV application in biofouling environments, maintaining sensitivity comparable to PEDOT:Nafion CFs and superior to uncoated CFs in the short term. However, during extended passive exposure, their performance declined to levels similar to uncoated CFs and lower than PEDOT:Nafion coated CFs. Together, these findings suggest that the stability of B‐NCD CFs is primarily associated with their mechanical robustness and resistance to repeated electrochemical cycling rather than intrinsic biofouling resistance.

**FIGURE 8:**
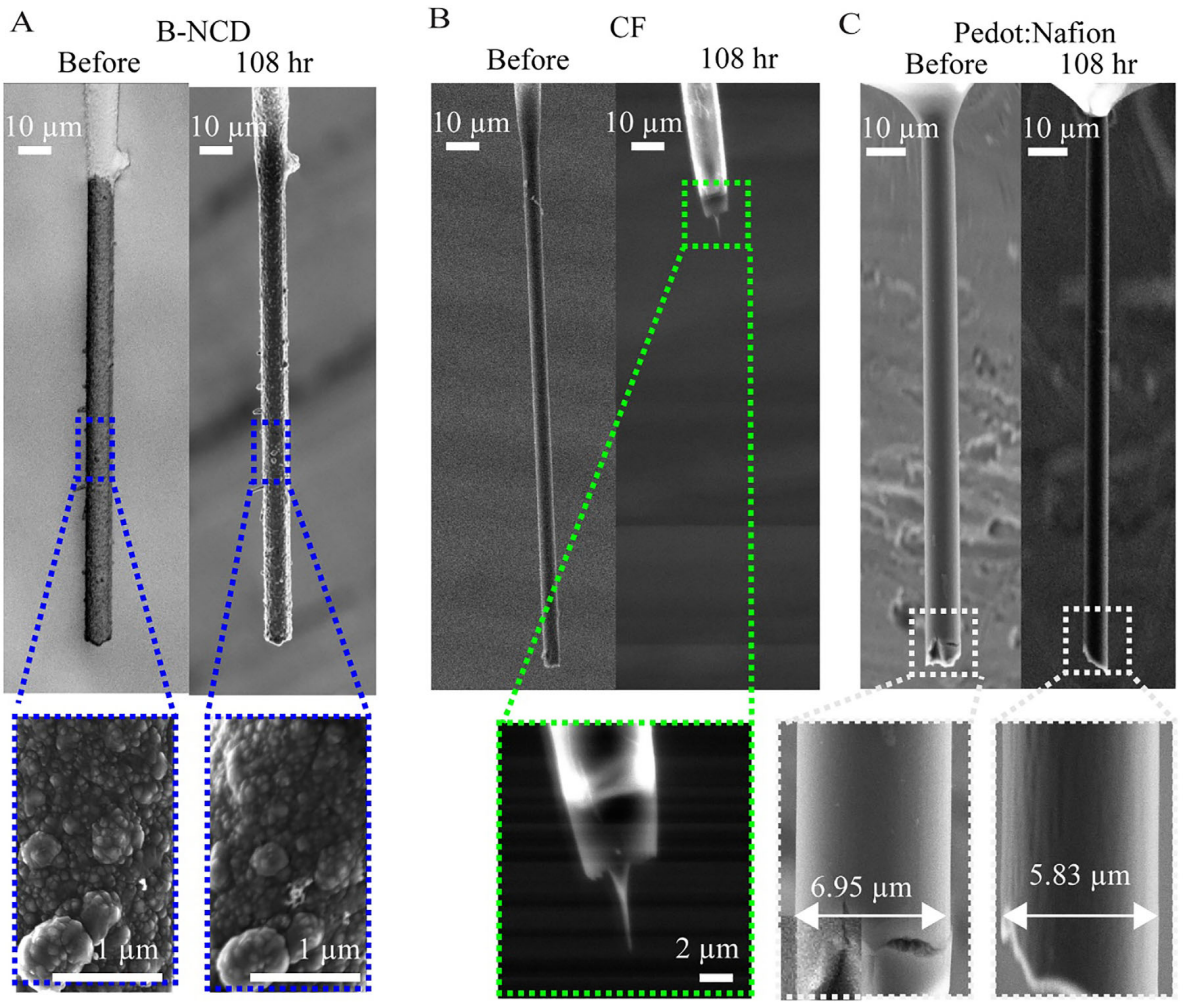
SEM images of representative B‐NCD coated (A), uncoated (B), and PEDOT:Nafion coated (C) CF electrodes before and after 108 h of simulated repeated FSCV cycling. No degradation is observed with the B‐NCD coating. The uncoated CF electrode showed complete degradation of the electrode tip. PEDOT:Nafion coating showed signs of bulk etching of the fiber over the same time period.

To assess the electrochemical stability of all three electrode types under repeated potential cycling over time, without the confounding effects of biofouling from protein or chemical adsorption, FSCV waveforms (−0.4 to +1.5 V, scan rate of 400 mV/s, 60 Hz) were repeatedly applied while the electrodes were submersed in PBS. This higher potential (+1.5 vs +1.0 or +1.3 V) was selected to mimic the surface renewal process often required in repeated FSCV applications. Such higher potentials are also necessary for the electrochemical detection of neurochemicals beyond dopamine, such as adenosine at 1.5 V [84]. These results clearly demonstrate substantial degradation of the CF after a period of 36 h (Figure S4), with complete degradation of the CF electrode (*n* = 3) occurring after a 108‐h (Figure 8B). PEDOT:Nafion coated CFs also showed signs of degradation (Figure 8C), etching around 1 µm over the 108‐hr period (*n* = 4). The B‐NCD electrodes, however, showed no evidence of degradation after the same time period (*n* = 6) (Figure 8A; Figure S5). One sample tested that did not have uniform growth around the fiber exhibited etching in areas where CF was not covered (Figure S5), whereas the areas covered with B‐NCD showed no signs of degradation, highlighting the robustness of the coating.

The EIS spectrum of the B‐NCD coated CF showed no significant difference (paired *t*‐test, *p* = 0.103, *n* = 5) before and after cycling for 108 h, with the impedance at 1 kHz showing a slight reduction from 428 ± 234 to 291 ± 229 kΩ (Figure S6). This is a positive indication of the electrochemical robustness of the coating. The background currents of the representative electrodes before, after 36 and 108 h can be seen in Figure S7 for the CF and B‐NCD electrodes, respectively. As expected, both electrodes exhibited an increase in background current after an initial period due to the initial cleaning and activation of the electrode surface [11]. This activation appears to remain constant in the B‐NCD electrode as the background current is similar from the 36 to 108 h cycling period. This suggests the treatment waveform has activated the surface without any of the bulk removal that would be expected in conventional CF electrodes. The decrease in the background current for the CF electrode from 36 to 108 h is a clear indicator of the bulk degradation of the electrode. The B‐NCD electrodes were tested for their dopamine sensitivity before and after cycling (Figure S8). No significant difference (paired *t*‐test, *n* = 5) was found before (*p* = 0.36) and after (*p* = 0.08) normalization of the electrodes, further indicating the electrochemical robustness of the coating. There was also no significant increase (*p* = 0.07) in the background currents of these electrodes before and after, indicating that the electrodes remained stable. Future experiments into the complete performance of a chronically implanted B‐NCD electrode for dopamine detection and further investigation into what is occurring at the surface of the coating over time is the logical extension of this work.

### 3.6 Biocompatibility Assessment

**FIGURE 9:**
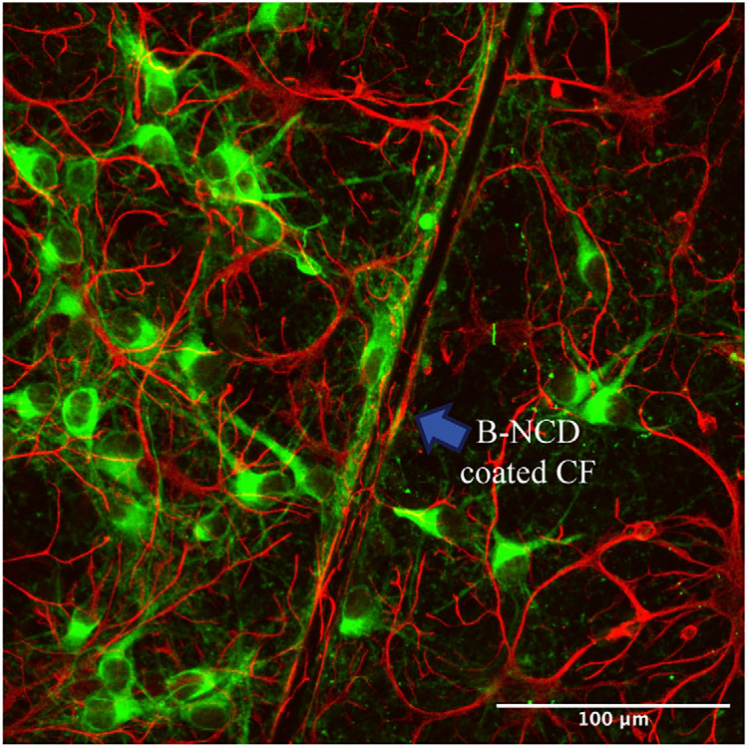
Representative fluorescence microscopy image of neural cells at day 7 in vitro. The blue arrow indicates the position of a B‐NCD coated CF. Neurons (βIII tubulin, green) and astrocytes (GFAP, red) showed successful adhesion and growth on and around the coated CF.

Rat postnatal cortical cells were seeded onto the B‐NCD coated CFs, and cultures were fixed and immunostained for neuronal and astrocyte markers at 7 days in vitro. Immunohistochemical results (Figure 9) indicate that the B‐NCD coated CF supported neural cell attachment and neurite outgrowth. Primary neurons, labelled with βIII tubulin (green), and astrocytes, labelled with GFAP (red), were clearly observed growing on and adjacent to the implanted B‐NCD coated CF. These findings are consistent with previous reports indicating that both diamond and CF are biocompatible and can support, or even enhance, neural cell adhesion and growth [41, 85, 86]. Our previous study suggested that the improved neuronal survival on conductive diamond surfaces was associated with the upregulation of genes involved in cell adhesion and gap junction formation, one key component in neuronal communication.

### 3.7 Mechanical Testing

**FIGURE 10:**
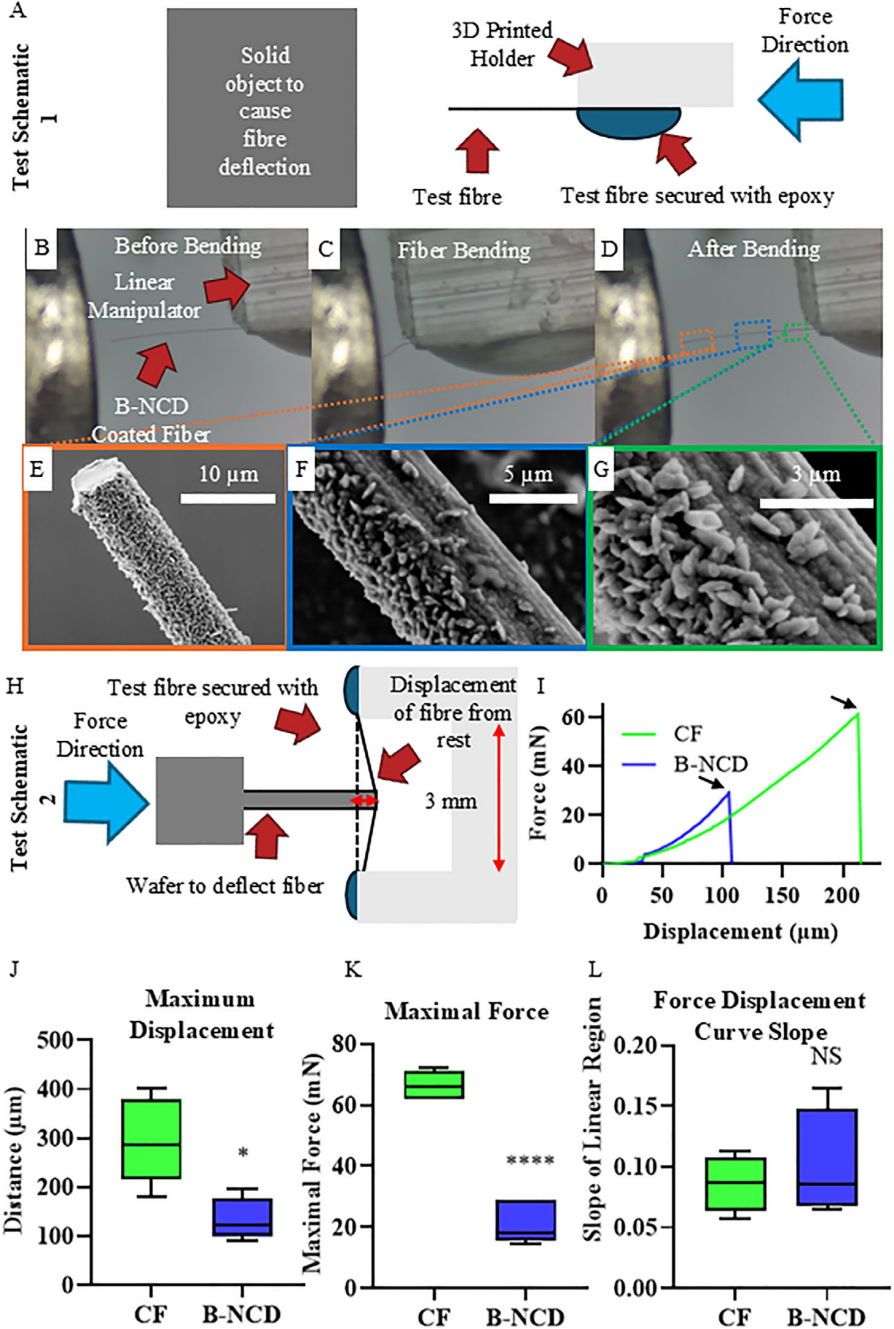
Mechanical testing of the B‐NCD coated CFs by driving them into a solid object (A) showing the fiber at rest (B), the fiber bent to 25% of its original length (C), and the fiber after the test (D). SEM images of the tip of the fiber (E) and various positions down the length of the fiber (F,G) show no evidence of fiber or coating damage. The schematic of the three‐point bending test (H) wherein uncoated CFs and B‐NCD coated fibers were attached at two ends to a 3D printed holder and pressed against a force gauge. The force‐displacement curves of representative fibers are shown in (I), with arrows indicating the failure point of the fiber. The displacement of the fiber from rest (J), maximal force before mechanical failure (K), and the slope of the initial linear region of the force‐displacement curves (H) are shown. (*n* = 6, ^*^ *p*<0.05, ^****^ *p*<0.001, NS not significant.

To evaluate the durability of the fibers after coating, B‐NCD coated fibers were attached to a linear manipulator and pressed against a force gauge (Figure 10A). The fibers were bent to 25% of their original length and then retracted (Figure 10B,C). SEM images after testing indicated no cracks or damage to the coating or fiber (Figure 10D–G). These results are important as previous work on diamond coated electrodes showed reduced flexibility and coating cracks, negating the CF's mechanical properties that make it promising as a neural electrode material [57]. The bending was repeated for 100 cycles (Figure S9) to determine the fatigue failure of the coating, with no obvious sign of delamination or breakage (*n* = 21).

Fibers were also subjected to shear force testing to determine the maximal force that the fiber could endure when bent whilst fixed at both ends (Figure 10H). The displacement and force were measured to determine how far a 3 mm long fiber could be bent before mechanical failure (Figure 10I). The B‐NCD coated CFs showed a statistically significant reduction in both displacement and maximal force experienced before failure (Figure 10J,K). The uncoated CF was able to be compressed 295 ± 78 µm, whereas the B‐NCD coated could only withstand a deflection of 133 ± 38 µm (n = 6, unpaired student *t*‐test, p = 0.012). The corresponding maximal forces before failure were 66.5 ± 4.5 mN for uncoated CFs and 20.7 ± 6.0 mN for the B‐NCD coated fibers (unpaired student *t*‐test, *p*<0.001).

The displacement *d* under a lateral force *F* follows the equation:

$$d=\frac{L^{3}F}{48IE}$$

where *L* represents the fiber length, *E* represents the bending modulus of elasticity, and *I* is the area moment of inertia of the fiber cross‐section.

Successful insertion of the fibers requires the insertion force to be lower than the critical buckling force, *F(buckling)*, which is given by:

$$F\;\left(buckilng\right)=\frac{\pi^{2}IE}{{\left(KL\right)}^{2}}$$

where *K* is the effective length factor.

This buckling force was too small to be reliably measured using our force gauge. However, for fibers of the same length, *F(buckling)* is directly proportional to the slope *F/d* in the initial linear region of the force‐displacement curve (as shown in Figure 10I), which reflects the fiber's resistance to bending. The average value of the slope was higher for B‐NCD fibers (Figure 10L), but the difference compared to the uncoated CFs was not statistically significant (unpaired student *t*‐test, *p* = 0.591). This finding supports that the B‐NCD coating would be suitable for implantation into neural tissue.

### 3.8 In Vivo Validation

**FIGURE 11:**
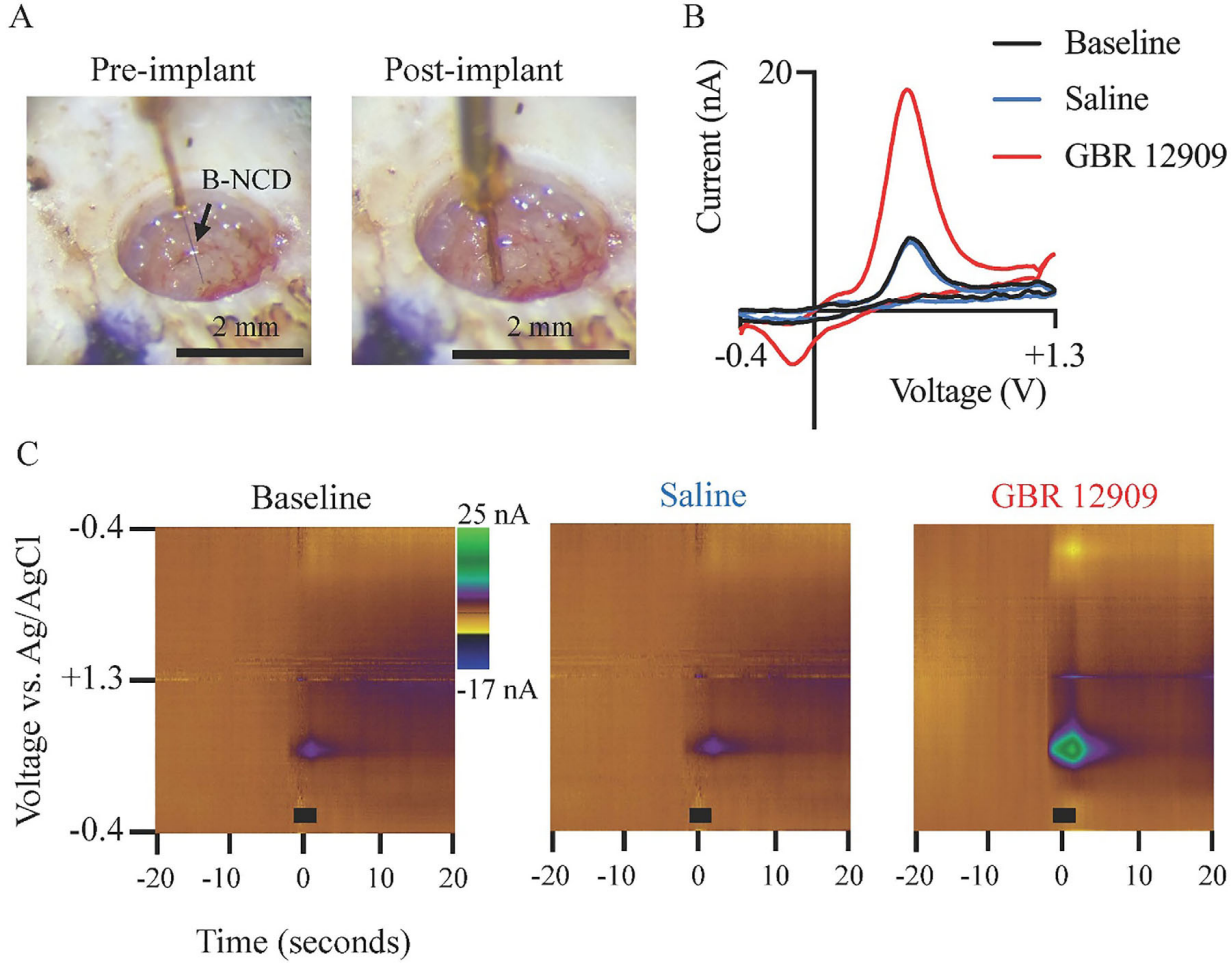
In vivo validation of B‐NCD coated CF electrodes. (A) Photographs of a trephine burr hole in the rodent skull before and after stereotaxic implantation of the B‐NCD CF into the nucleus accumbens. (B) Background‐subtracted dopamine voltammograms at the time of peak response at baseline (black), with saline (blue), and 45 min after GBR‐12909 administration (red). (C) Representative FSCV pseudocolor plots showing redox current generated at potentials characteristic of dopamine with each experimental intervention. The black bar indicates the duration (3s) of stimulation.

To evaluate the coating's functional applicability for in vivo dopamine detection, a B‐NCD coated CF electrode was implanted into the rodent nucleus accumbens to record phasic dopamine release with FSCV following electrical stimulation of the ventral tegmental area (Figure 11A). Vehicle (saline) administration did not alter evoked peak oxidative current relative to baseline (baseline = 5.80 nA, saline = 5.72 nA). In contrast, 45 min after systemic GBR 12909, the amplitude of evoked dopamine increased to a peak value of 18.18 nA (Figure 11B,C). These findings constitute a single‐animal (*n* = 1), proof‐of‐concept validation that demonstrates that the B‐NCD coated CF can reliably detect phasic dopamine in vivo and is sensitive to pharmacologically induced increases in extracellular dopamine.

## 4 Conclusion

We introduced an innovative method for fabricating CF electrodes uniformly coated with B‐NCD for phasic dopamine sensing. These electrodes leverage the small scale of the CF electrodes and the electrochemical stability and mechanical robustness of the B‐NCD coating. Unlike prior research utilizing diamond‐based biosensors, our results demonstrate that B‐NCD coated CF electrodes, when applied in FSCV for phasic dopamine detection, exhibit sensitivity levels on par with both uncoated and PEDOT:Nafion coated CFs. Moreover, in vitro experimentation employing BSA to simulate a biofouling environment reveals that when repeatedly cycled using FSCV, B‐NCD electrodes maintained improved dopamine sensing stability compared to uncoated and PEDOT:Nafion coated CFs. Notably, the B‐NCD coated fibers withstood repeated FSCV cycling under voltage ranges that degrade the uncoated and PEDOT:Nafion coated CFs, highlighting their superior electrochemical robustness.

In addition to their stability, the B‐NCD coated fibers exhibited mechanical flexibility and robustness suitable for chronic neural implantation. The B‐NCD electrodes leverage the electrical, chemical, and biocompatibility properties of a diamond coating, whilst mitigating mechanical failure risks common to more brittle diamond structures. Cell culture experiments confirmed that the B‐NCD surface supports neuronal growth, further validating its biocompatibility. Finally, in vivo recordings validated the mechanical robustness and functional performance of the B‐NCD electrodes, demonstrating their feasibility for use in preclinical experimentation utilizing FSCV to monitor phasic dopamine fluctuations.

These findings suggest that B‐NCD coated CF electrodes are well‐suited for chronic FSCV applications. Further experiments exploring the effect of the boron dopant concentration to tune the “diamond‐like” and “graphitic‐like” properties of the coating could allow for the development of application‐specific electrodes with optimized mechanical and electrical properties. Future work will focus on optimization of these electrodes for chronic dopamine detection using FSCV and alternative voltametric techniques, including those used for tonic dopamine measurement. Future work will also involve investigating their chronic in vivo biocompatibility and stability in these sensing experiments. Such efforts will help establish the full potential and utility of these electrodes for chronic in vivo experimentation, advancing our understanding and treatment of neurological disorders.

## Conflicts of Interest

The investigators associated with this project and Mayo Clinic have a financial conflict of interest in the technology used in the research, in that they may stand to gain financially from the successful outcome of the research. SH is a former employee of Carbon Cybernetics Pty Ltd., a company developing diamond and carbon‐based medical devices. MRI, DJG, and SP are shareholders and/or public officers of Carbon Cybernetics Pty Ltd.

## Supporting information




**Supporting file**: adhm70611‐sup‐0001‐SuppMat.docx

## Data Availability

The data that support the findings of this study are available from the corresponding author upon reasonable request.
