# Supplementary material for: Boron‐Doped Nano‐Crystalline Coated Carbon Fibers for Phasic Dopamine Sensing
Source: Adv Healthc Mater. 2025 Dec 18;15(15):e03945. doi: 10.1002/adhm.202503945 (PMC13088755; doi:10.1002/adhm.202503945)
Supplement: Supplementary file 1 — Supporting file: adhm70611‐sup‐0001‐SuppMat.docx [file ADHM-15-0-s001.docx]

**Boron-doped nano-crystalline coated carbon fibers for phasic dopamine sensing**

Simon J. Higham^1,2+^, Juan M. Rojas Cabrera^3,4+^, Youngjong Kwak^,4,5^, Lydia Hong^4^, Andre Chambers^2^, Athavan Nadarajah^2^, Sorel E. De Leon ^1^, Young Jun Jung^2,6^, Negin Jalilinejad^2^, Charles Blaha^4^, Dong Pyo Jang^5^, Yoonbae Oh^4,7^, Kendall Lee^4,7^, Alastair Stacey^1^, Shaun L. Cloherty^1^, Michael R. Ibbotson^6^, David J. Garrett^1^, Steven Prawer^2^, Hojin Shin^4,7,*^, Wei Tong^2,8*^

^1^ School of Engineering, RMIT University, Melbourne, VIC, Australia

^2^ School of Physics, The University of Melbourne, Melbourne, VIC, Australia

^3^ Medical Scientist Training Program, Mayo Clinic, Rochester, MN, USA

^4^ Department of Neurologic Surgery, Mayo Clinic, Rochester, MN, USA

^5^ Department of Biomedical Engineering, Hanyang University, Seoul, Korea

^6^ School of Biomedical Engineering, The University of Melbourne, Melbourne, VIC, Australia

^7^ Department of Biomedical Engineering, Mayo Clinic, Rochester, MN, USA

^8^ The Graeme Clark Institute, The University of Melbourne, Melbourne, VIC, Australia


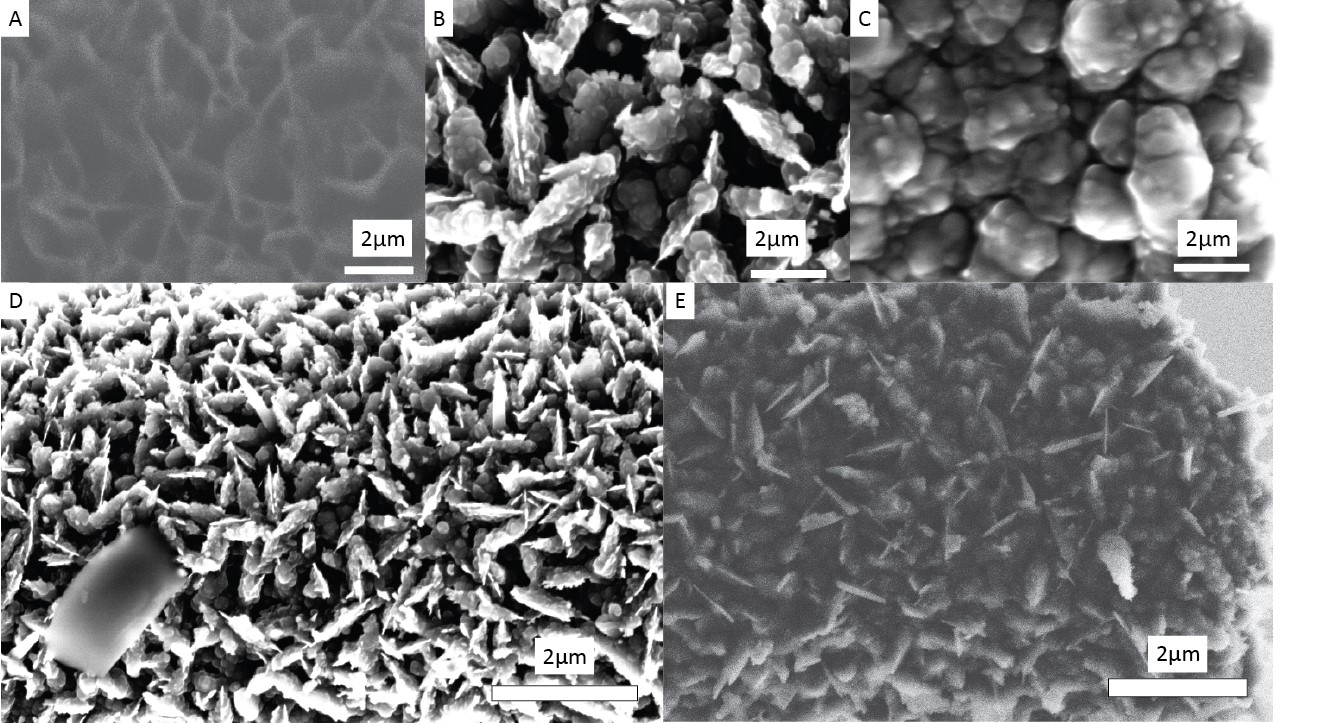


**Figure S1.** ((SEM Images showing the variance within the growth bundle. Complete CNW (A), partial CNW with some small crystals (B) and completely nanocrystalline coating (C).))


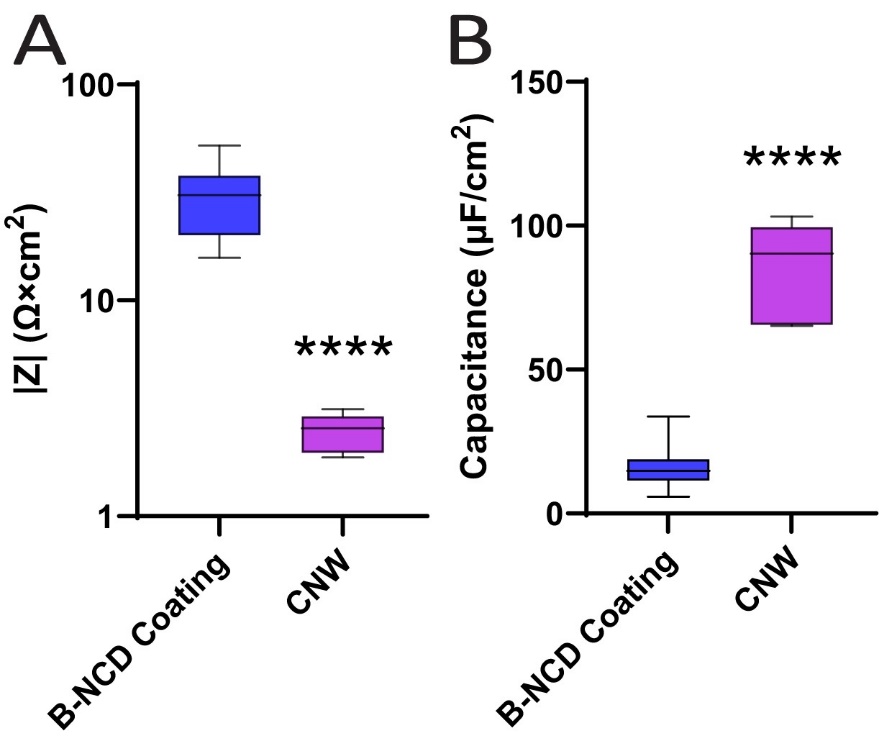


**Figure S2.** ((Normalized impedance magnitude at 1kHz (A) of electrodes with complete B-NCD (32.09 ± 11.09 Ω×cm2) and predominately CNW coatings (2.459 ± 0.500 Ω×cm2). Specific Capacitance (B) of electrodes with complete B-NCD (16.1 ± 6.81 µF/cm2) and predominately CNW coatings (84.01 ± 17.30 µF/cm2). (n=12 and n=6, respectively, **** p<0.001)))

**
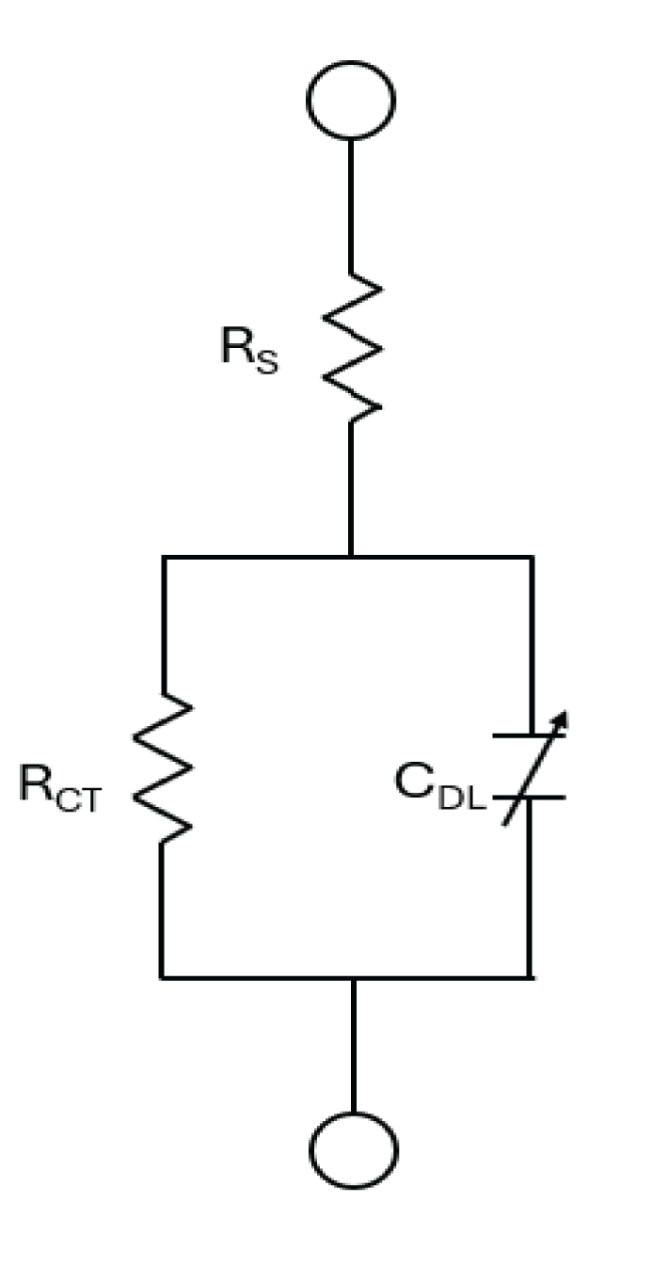
**

**Figure S3.** ((The Randles circuit model used to estimate the specific capacitance.))


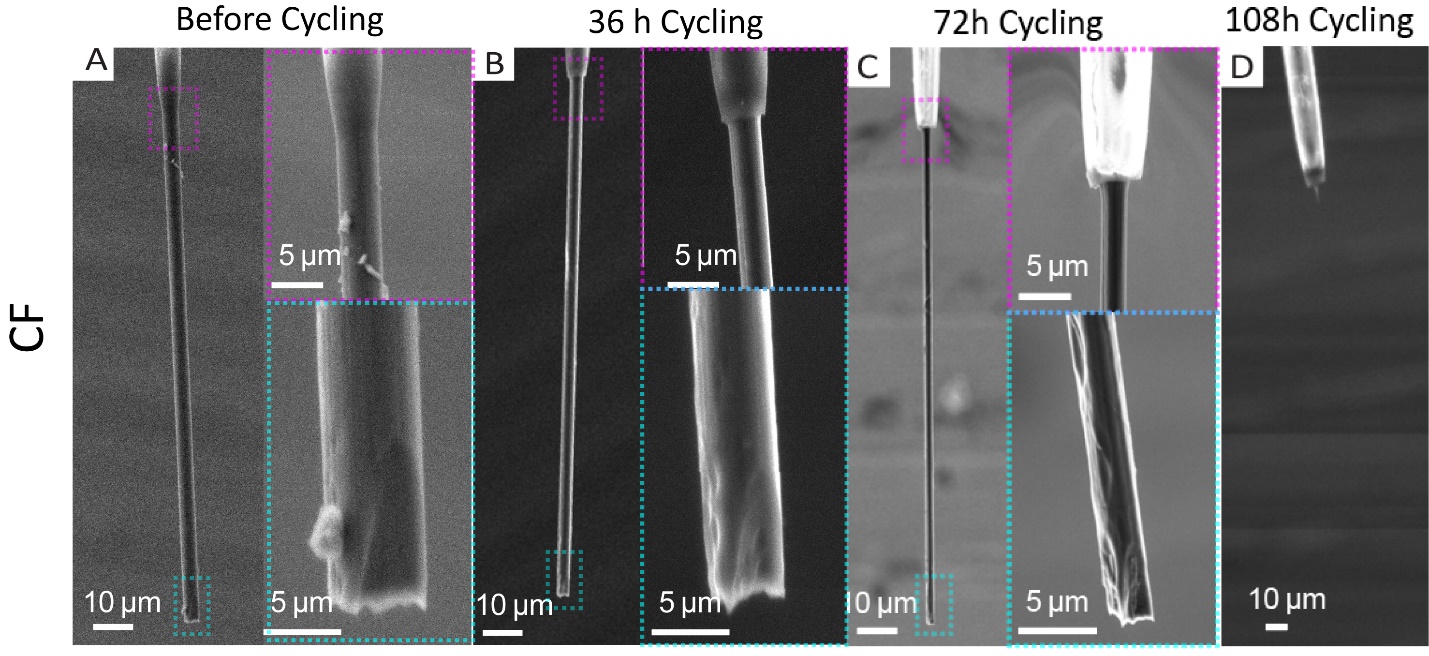


**Figure S4.** ((SEM Images of one representative CF electrode before (A), after 36 hours (B), 72 hours (C) and 108 hours (D) of FSCV cycling.))


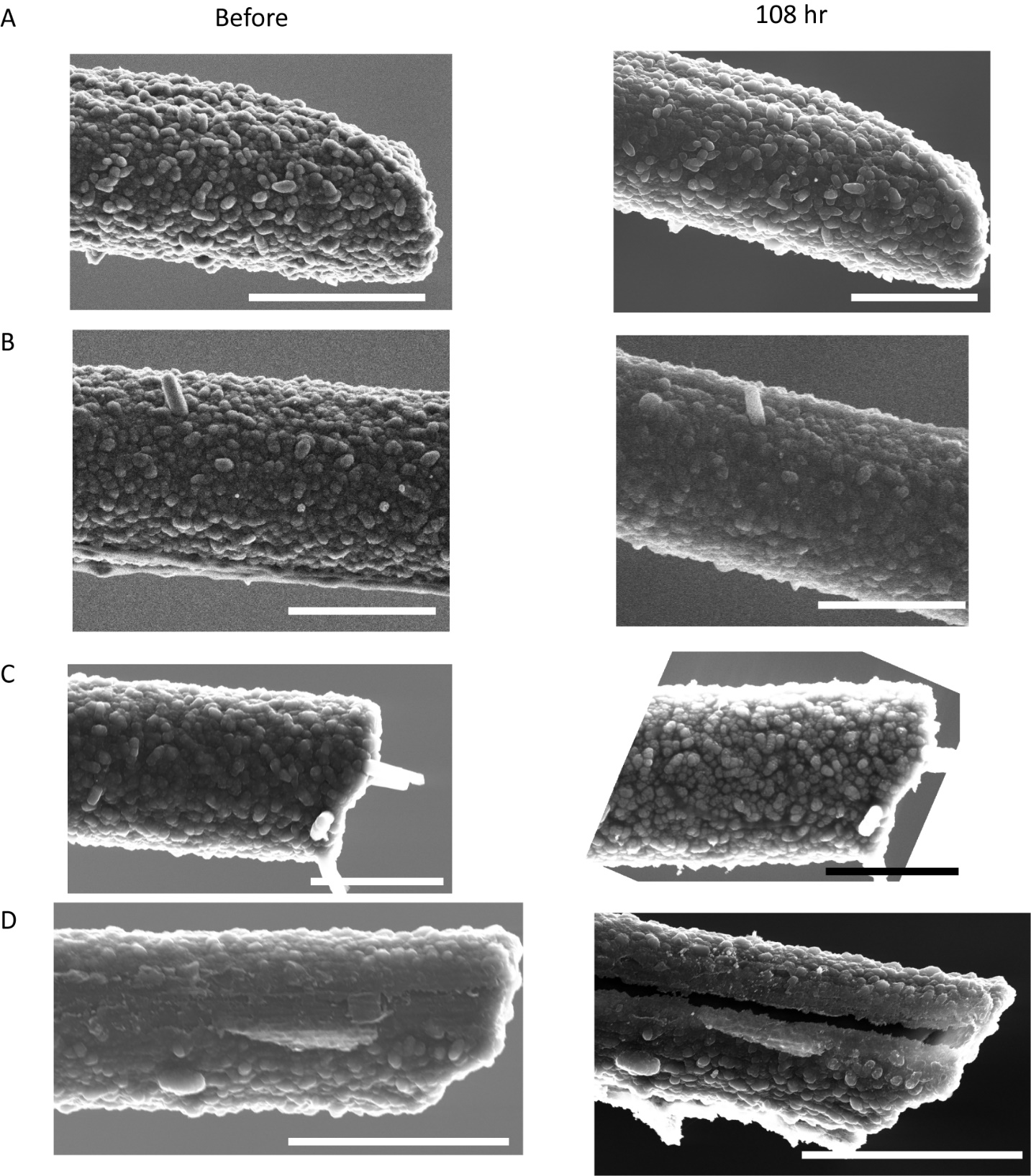


**Figure S5.** ((Other representative samples of B-NCD coated CF before and after cycling showing no degradation (A-C). One sample that exhibited non uniform B-NCD coating and clear etching in the regions where the non-uniformity occurred (D) (Scale bar 10 µm).))


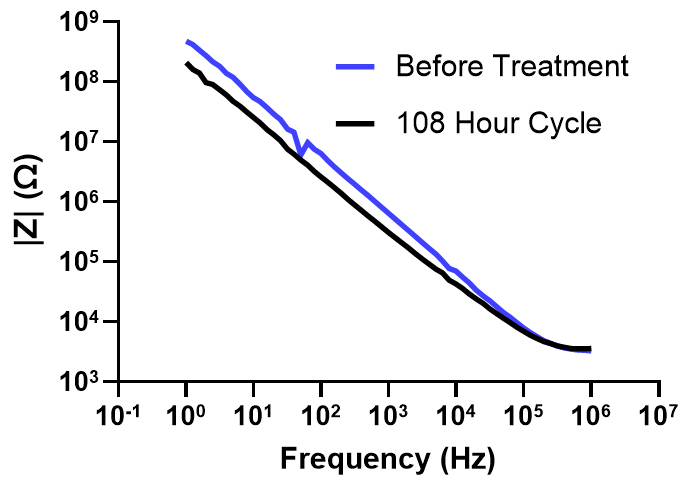


**Figure S6.** ((Impedance spectroscopy of a B-NCD electrode before and after 108-hour FSCV cycling.))


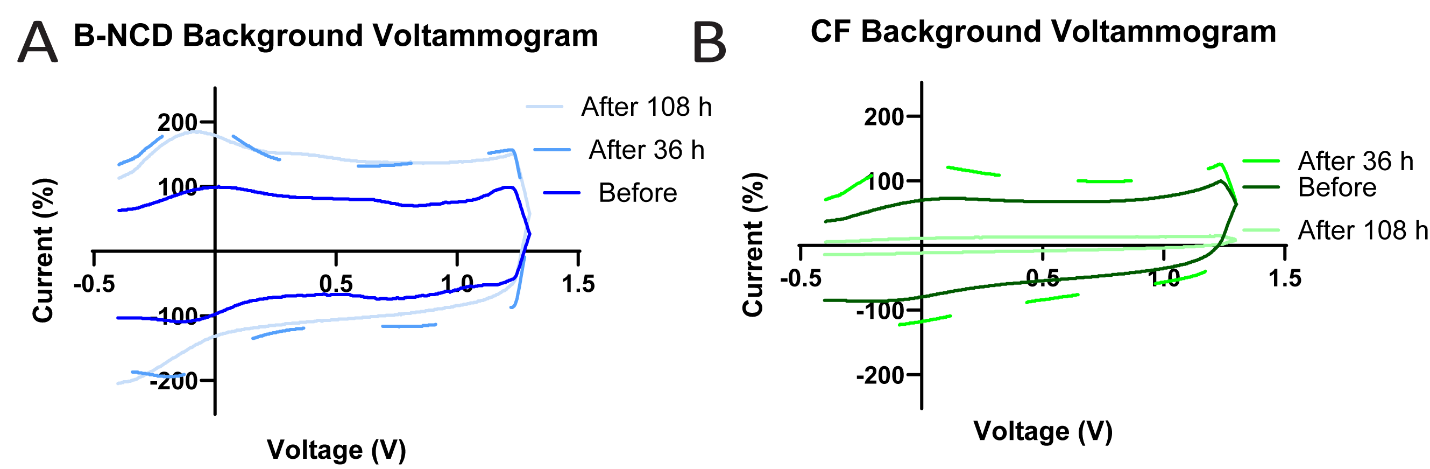


**Figure S7.** ((Background currents of representative electrodes of B-NCD (A) and CF (B) before, after 36-hour and after 108-hour FSCV cycling, normalized to the maximum current in the before voltammogram for each sample to show the change from baseline as a result of repeated cycling.))


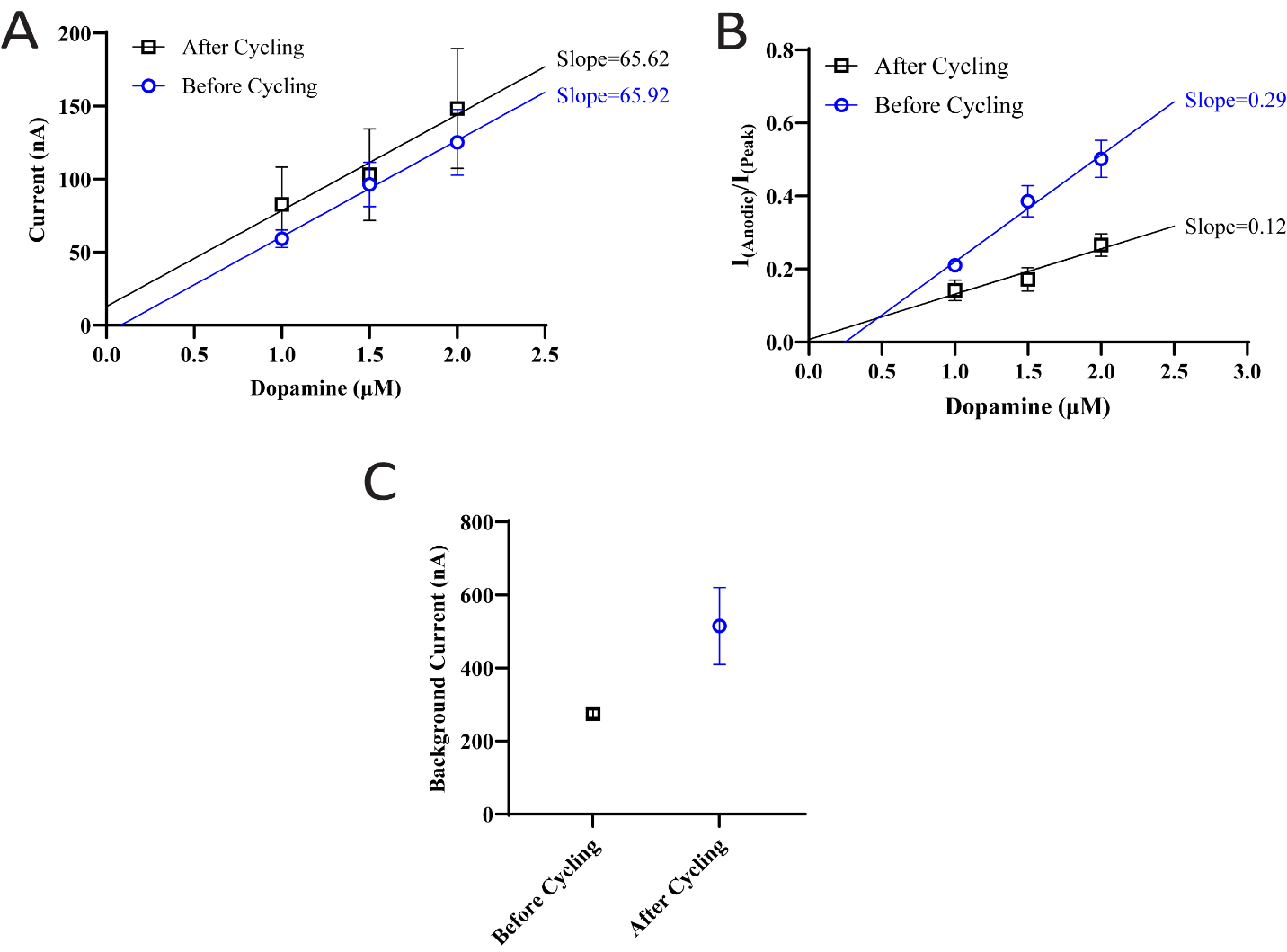


**Figure S8.** ((In vitro dopamine sensing experiments conducted on B-NCD fibers before and after cycling experiments along with linear regression fitting (n=5). No significant difference is found between the samples with (A)(p=0.36) and without (B)(p=0.08) current normalization. The background currents for the electrodes are shown in C before and after cycling (ns, p=0.07).))


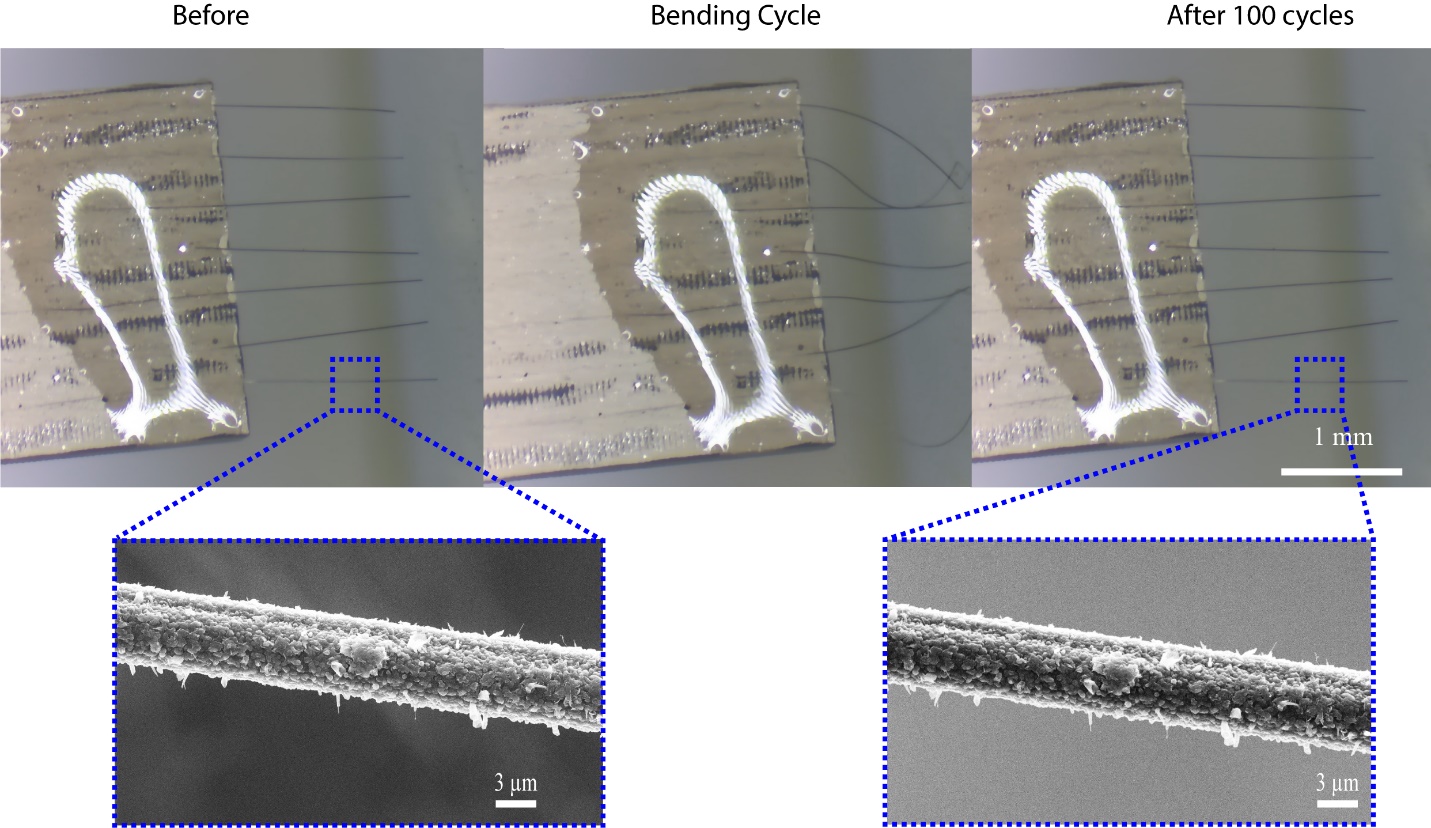


**Figure S9.** ((Repeated bending tests (100 cycles) to demonstrate the resistance of the coating to mechanical fatigue, with a representative SEM image taken before and after. No evidence (Optical or SEM) showed any sign of wear on the fiber or coating.))


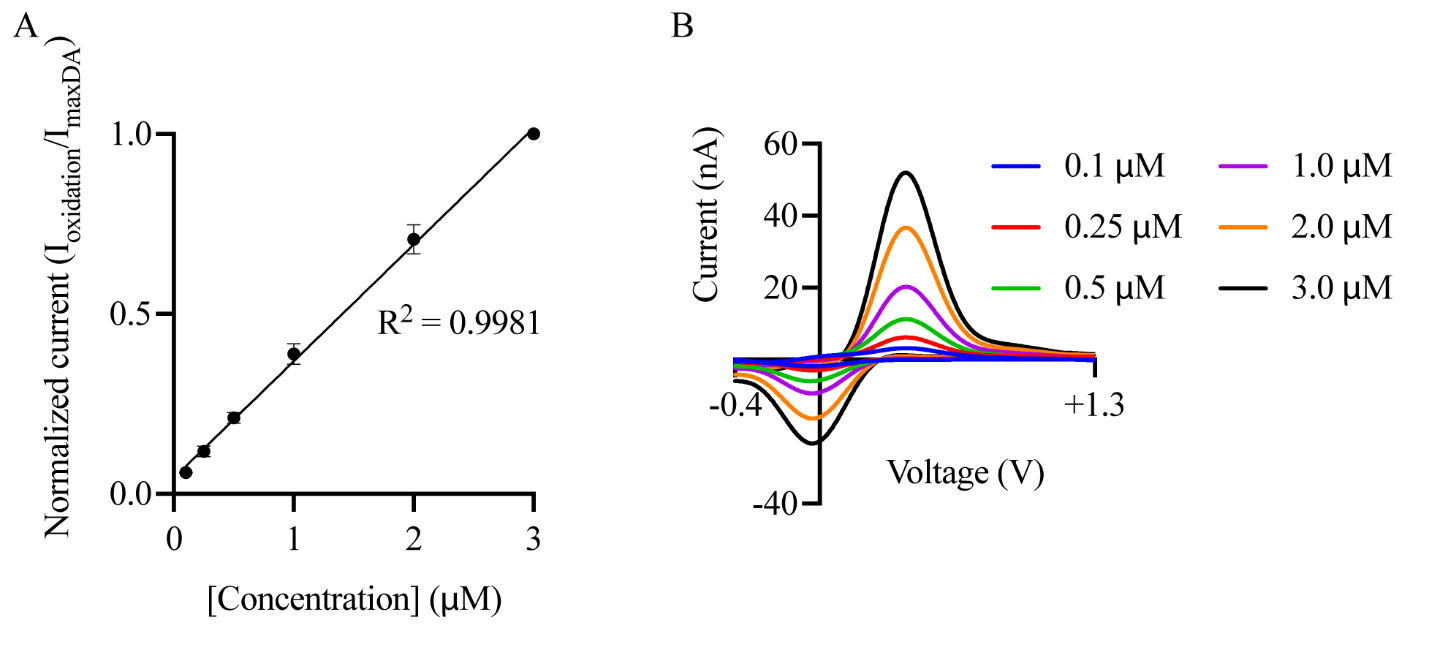


**Figure S10.** ((Dopamine detection calibration curve for B-NCD coated CF electrodes (A). B-NCD electrodes (n = 3) were challenged with the following concentrations of dopamine into Tris buffer solution: 0.1, 0.25, 0.5, 1.0, 2.0, and 3.0 µM. Data was normalized by dividing the peak oxidation current at each dopamine concentration by the peak oxidation current for the maximum dopamine concentration tested. Application of a linear regression fit provided an R2 value of 0.9981 (A). Representative background-subtracted dopamine voltammograms showing distinct peaks at potentials where dopamine is oxidized (~+0.6V) and reduced (~-0.2V) are shown for each concentration tested. Normalized current is shown as mean percentage of the maximum oxidation current ± standard deviation (SD)(B).))
